# Genomic prediction of the recombination rate variation in barley – A route to highly recombinogenic genotypes

**DOI:** 10.1111/pbi.13746

**Published:** 2021-12-11

**Authors:** Federico Casale, Delphine Van Inghelandt, Marius Weisweiler, Jinquan Li, Benjamin Stich

**Affiliations:** ^1^ Institute of Quantitative Genetics and Genomics of Plants Heinrich Heine University Düsseldorf Germany; ^2^ Max Planck Institute for Plant Breeding Research Köln Germany; ^3^ Cluster of Excellence on Plant Sciences From Complex Traits Towards Synthetic Modules Düsseldorf Germany; ^4^ Strube D&S GmbH Söllingen Germany

**Keywords:** recombination rate, genomic prediction, GBLUP, plant breeding

## Abstract

Meiotic recombination is not only fundamental to the adaptation of sexually reproducing eukaryotes in nature but increased recombination rates facilitate the combination of favourable alleles into a single haplotype in breeding programmes. The main objectives of this study were to (i) assess the extent and distribution of the recombination rate variation in cultivated barley (*Hordeum vulgare* L.), (ii) quantify the importance of the general and specific recombination effects, and (iii) evaluate a genomic selection approach’s ability to predict the recombination rate variation. Genetic maps were created for the 45 segregating populations that were derived from crosses among 23 spring barley inbreds with origins across the world. The genome‐wide recombination rate among populations ranged from 0.31 to 0.73 cM/Mbp. The crossing design used in this study allowed to separate the general recombination effects (GRE) of individual parental inbreds from the specific recombination effects (SRE) caused by the combinations of parental inbreds. The variance of the genome‐wide GRE was found to be about eight times the variance of the SRE. This finding indicated that parental inbreds differ in the efficiency of their recombination machinery. The ability to predict the chromosome or genome‐wide recombination rate of an inbred ranged from 0.80 to 0.85. These results suggest that a reliable screening of large genetic materials for their potential to cause a high extent of genetic recombination in their progeny is possible, allowing to systematically manipulate the recombination rate using natural variation.

## Introduction

The reciprocal genetic exchange between homologous chromosomes is termed crossover (CO), and it is required for the proper chromosomal segregation during the first meiotic division (Morgan, 1916). This genetic reshuffling in addition to the independent segregation of chromosomes enables meiosis to produce new allelic combinations in the resulting gametes. This process is called meiotic recombination. Consequently, the rate and distribution pattern of recombination events along the genome determine the effectiveness of selection in removing deleterious mutations and increasing the frequency of beneficial allele combinations (Henderson, 2012). This makes meiotic recombination a fundamental element not only for the adaptation of sexually reproducing eukaryotes in nature but also for stacking many favourable alleles into a single haplotype in breeding schemes (Nachman, 2002; Tiley and Burleigh, 2015). The manipulation of the factors influencing the rate and distribution of recombination events along the genome, therefore, has the potential to accelerate plant and animal breeding (Choi and Henderson, 2015).

Studies on model plants have increased our knowledge about the mechanism and regulation of recombination considerably (for review, see Mercier *et al*., 2015; Wang and Copenhaver, 2018). While this has opened up possibilities for the manipulation of genetic recombination by environmental factors such as temperature (Arrieta *et al*., 2020; Higgins *et al*., 2012), an even higher impact is expected from approaches that rely on altering the genetics of recombination (Taagen *et al*., 2020). The use of genome‐editing approaches that induce double‐stranded breaks (DSBs) or modify epigenomes at the desired sites of recombination (Hayut *et al*., 2017; Underwood *et al*., 2018), and the manipulation of CO factors (Mieulet *et al*., 2018; Sarno *et al*., 2017; Tam *et al*., 2011) are increasingly applicable for achieving this goal. However, such approaches still face technical challenges such as to the genotype‐specific efficiency of genetic transformation to be effectively applied (Altpeter *et al*., 2016; Hayta *et al*., 2019). In addition to technical challenges, a constraint for the adoption of gene‐edited crops is government regulation (Taagen *et al*., 2020). As an alternative to controlled recombination via genome editing, the utilization of natural variation remains a possible way to manipulate recombination in plants.

The meiotic recombination rate is known to vary within and among species (Nachman, 2002). Over the last years, several studies have examined the intraspecific variation of recombination rate in animals (Booker *et al*., 2017; Chan *et al*., 2012; Coop *et al*., 2008; Dumont *et al*., 2009; Fledel‐Alon *et al*., 2011; Hunter *et al*., 2016; Kong *et al*., 2010; Meznar *et al*., 2010; Petit *et al*., 2017; Sandor *et al*., 2012) and plants such as *Arabidopsis* (Kim *et al*., 2007; Salomé *et al*., 2012; Ziolkowski *et al*., 2017), maize (Bauer *et al*., 2013; McMullen *et al*., 2009; Rodgers‐Melnick *et al*., 2015), wheat (Darrier *et al*., 2017; Gardiner *et al*., 2019; Jordan *et al*., 2018), rice (Marand *et al*., 2019), cotton (Shen *et al*., 2019), and *Eucalyptus* (Gion *et al*., 2016), which showed high extent of variation in the frequency and distribution of recombination events across the genomes among genotypes of the same species. Until now, the recombination rate variation in barley (*Hordeum vulgare* L.) was examined in crosses between wild and cultivated barley (Dreissig *et al*., 2020), which may be due to the structural variants (SVs) between both genomes, thus not fully representative of intraspecific variation. In this sense, information about the intraspecific recombination rates for cultivated barley, which is an important crop species and a model for the Triticeae tribe, is lacking.

The most robust approach to assess the recombination rate variation is to examine the co‐segregation pattern of alleles at linked loci in populations with known pedigree relatedness (Petit *et al*., 2017; Salomé *et al*., 2012). The pedigrees that were examined in an animal genetic context were designed such that one female was mainly recombined with one male (e.g. Smeds *et al*., 2016; Weng *et al*., 2014). Similarly, the nested association mapping designs that were used for evaluating the recombination rate variation among plants consist of progenies derived from the crosses between a diverse set of genotypes and a common parent (Dreissig *et al*., 2020; Jordan *et al*., 2018; McMullen *et al*., 2009). Such pedigrees, however, do not allow to assess whether a high recombination rate in the progenies is due to the general parental effect or from the specific combination of both parental genotypes. This information is crucial for designing experiments to alter the recombination rate systematically.

The idea to exploit the natural recombination rate variation to construct highly recombinogenic genotypes was proposed more than 30 years ago (Cederberg, 1985). Despite the dramatic advances in genotyping technology and the availability of new resources for genetic mapping (Beyer *et al*., 2008; Lee *et al*., 2002; Yu *et al*., 2008), such highly recombinogenic genotypes have not yet been developed in any crop species. This might be explained by the fact that the assessment of recombination properties requires considerable experimental efforts to generate and genotype one to several segregating populations for each accession of interest. Genomic selection approaches employ all available markers across the genomes to predict genotypic values and are nowadays used in most animal and crop breeding programmes because of their high prediction accuracy (Meuwissen *et al*., 2001). To our knowledge, no previous study has evaluated the potential of genomic selection (GS) to predict the genome‐wide and local recombination rate variation. These approaches may permit the development of highly recombinogenic lines in recurrent genomic selection programmes.

The main objectives of this study were to (i) assess the extent and distribution of recombination rate variation in cultivated barley, (ii) quantify the importance of the general and specific recombination effects, and (iii) evaluate a genomic selection approach’s ability to predict the recombination rate variation.

## Results

### Genetic variation and parental segregation among the DRR populations

**Figure 1:**
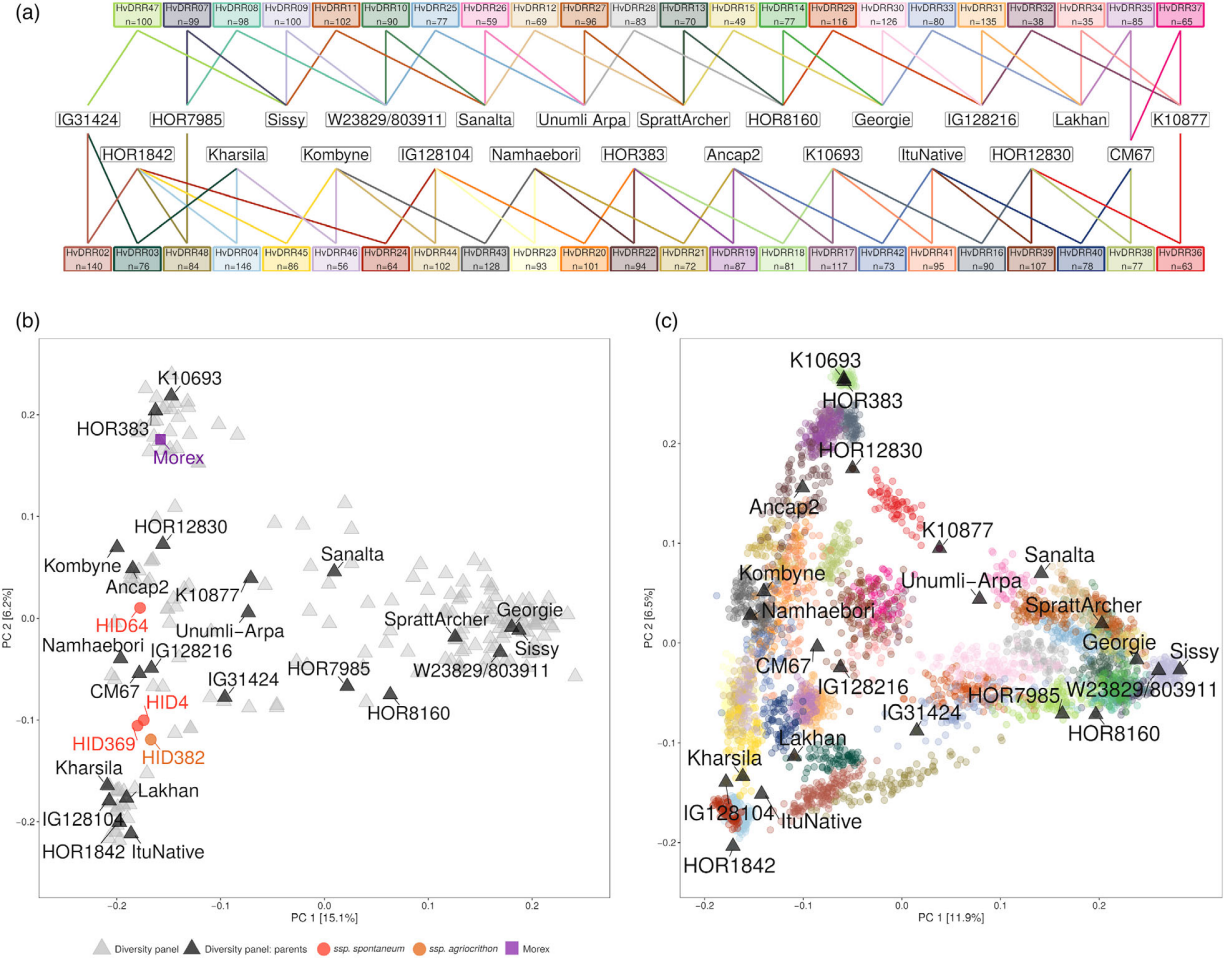
(a) The crossing scheme underlying the double round‐robin populations of barley. The number of recombinant inbred lines available per population is indicated below each population’s name. (b) Principal coordinate analysis of the diversity panel, Morex and three *ssp*. *spontaneum* and one *ssp*. *agriocrithon* accessions based on 36,077 SNP markers. PC 1 and PC 2 are the first and second principal coordinate, respectively, and the number in parentheses refers to the proportion of variance explained by the principal coordinates. (c) Principal coordinate analysis of the double round‐robin (DRR) populations and their parental inbreds based on 36,077 SNP markers. PC 1 and PC 2 are the first and second principal coordinate, respectively, and the number in parentheses refers to the proportion of variance explained by the principal coordinates. Parental inbreds are indicated by black triangles. Individuals from the same DRR population are indicated with dots of the same colour.

A principal coordinate analysis (PCoA) was performed for the diversity panel, three *ssp*. *spontaneum* and one *ssp*. *agriocrithon* accessions, and Morex (Figure 1b). The first axis separated the two rows from the six‐row genotypes, where the four wild barley accessions clustered with the latter. The result of the PCoA suggested that the parents of the double round‐robin (DRR) populations well represent the genotypic space of the diversity panel. In the PCoA of the DRR populations and their parental inbreds, the inbreds of each DRR population clustered together in between the position of their parental inbreds (Figure 1c), thereby illustrating the absence of pedigree errors. The assessment of segregation distortion (SD) demonstrated that for 38 of the 45 DRR populations, one or several genome regions were observed with an allele frequency that significantly (*P* < 0.05) deviated from 0.5. Several of the observed SD regions were found to be large with up to 300 Mbp (Figure S1). Interestingly, some of the DRR populations exhibited shared segregation trends depending on the parental inbred. Allelic segregation favoured the allele of IG128216 in chromosome 5H (50–350 Mbp) of the populations HvDRR30 and HvDRR31. Contrastingly, the allelic segregation on chromosome 6H (390–530 Mbp) of the populations HvDRR43, HvDRR44, HvDRR45, and HvDRR46 disfavoured the allele of Kombyne. Because the reported regions were long, many genes might have been responsible for the segregation bias.

### The intraspecific recombination variation in cultivated barley

The high‐density linkage maps that were constructed for the 45 DRR populations comprised 6,569–12,962 single‐nucleotide polymorphisms (SNPs) (Figure S2). This resulted in genetic maps with average distances between adjacent bins varying from 0.88 to 3.17 cM and the median of the longest gap across all populations being 23.99 cM. The median of Pearson’s correlation coefficient between the genetic and physical map position was 0.9 across all populations. This, together with the median fraction of 0.008 of the SNPs that were at a threshold of 5 cM non‐collinear to the physical map in each DRR population, indicated high collinearity between the obtained genetic maps and the reference genome.

**Figure 2:**
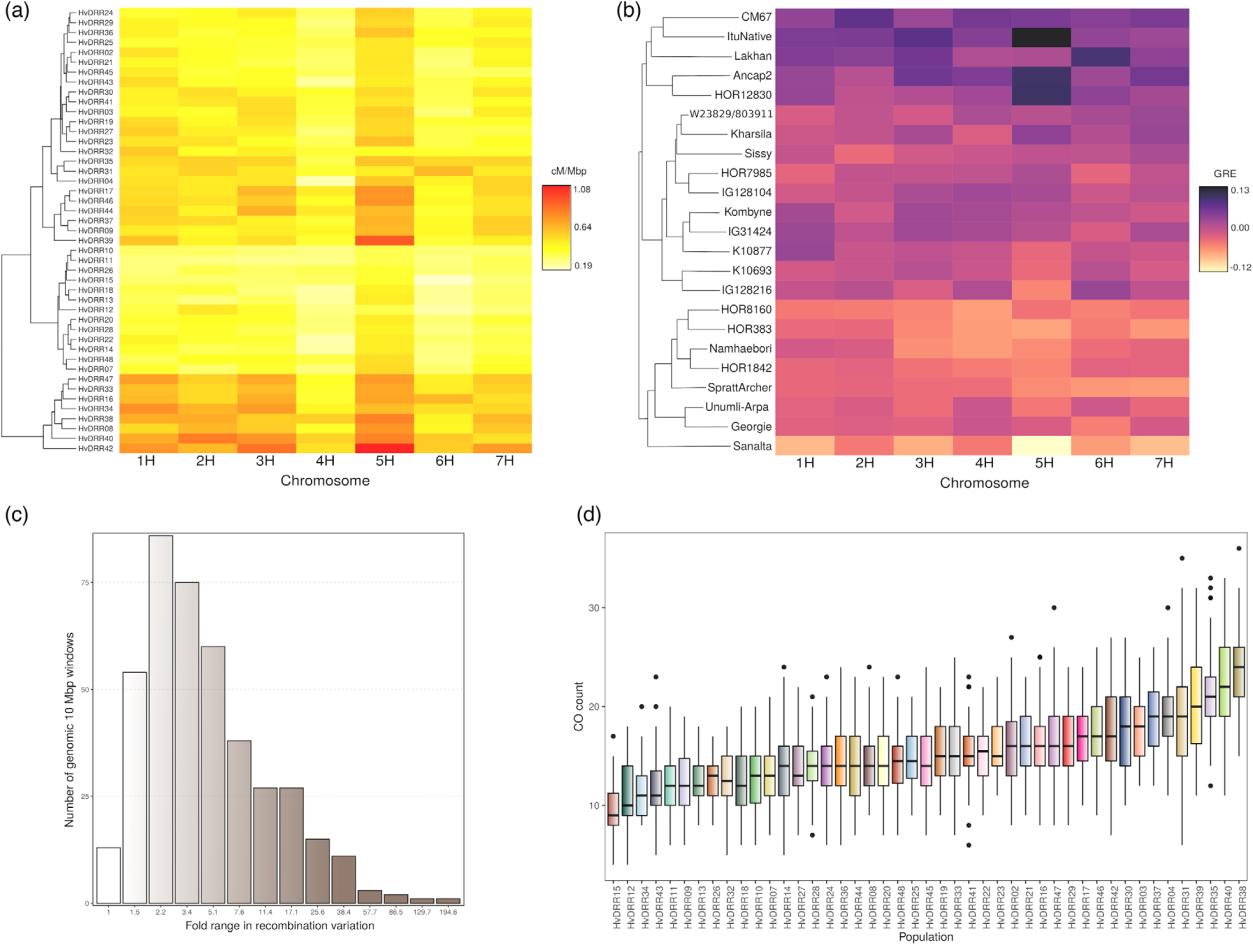
(a) Heat map of the chromosome‐wide recombination rates for the 45 double round‐robin populations. (b) Heat map of the chromosome‐wide phenotypic‐estimated general recombination effect (*GRE*_P_) for the 23 parental inbreds. Darker colours indicate higher recombination rates or *GRE*_P_ values. On the y‐axis, populations and inbreds are ordered according to their hierarchical complete clustering based on Euclidean distances of recombination rates and *GRE*_*P*_ values per chromosome respectively. (c) Histogram of the number of 10 Mbp windows by the fold range of recombination rate variation. (d) Boxplot of the number of counted genome‐wide crossovers (CO) for all DRR populations.

**Figure 3:**
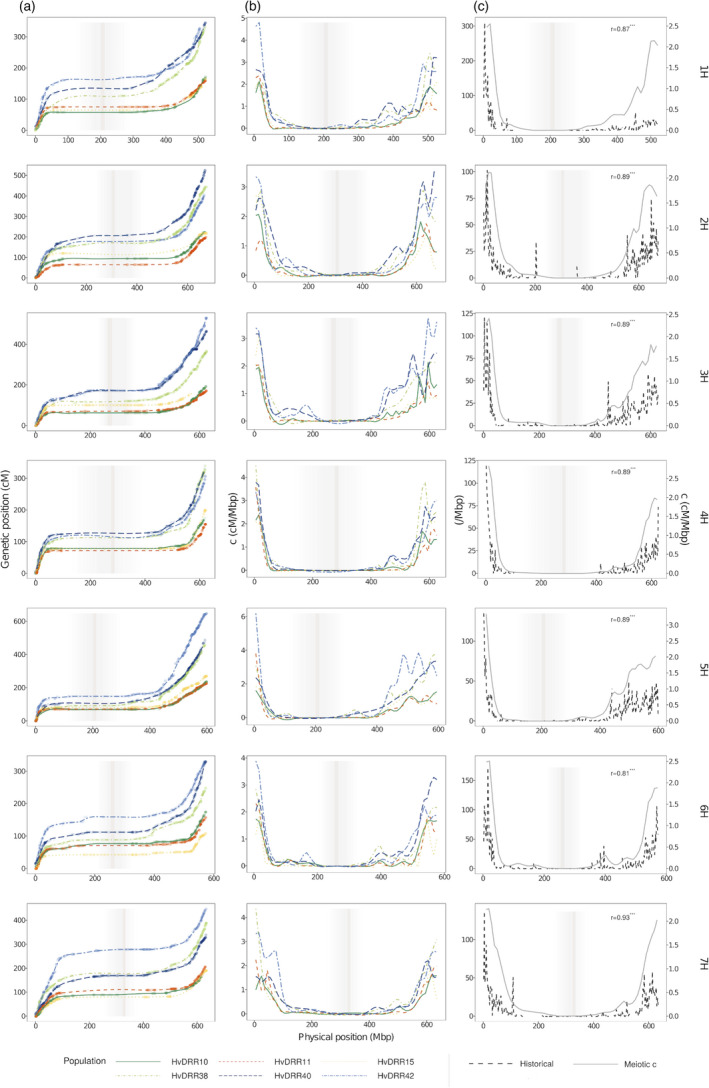
(a) Marey map and (b) meiotic recombination rate (*c*) landscape across the seven chromosomes of the three double round‐robin populations with the highest and lowest genomic recombination rates. (c) Historical recombination estimates *ρ_w_* (/Mbp, black‐dashed line) for the diversity panel and consensus meiotic recombination rate c (cM/Mbp, grey‐solid line) across all DRR populations along the seven barley chromosomes. The vertical line indicates the position of the centromere in the reference map and the shadow denotes the pericentromeric region. *r* is Spearman’s correlation coefficient between the historical recombination estimate ${\hat{\rho }}_{w}$ and the consensus (*c*) across 10 Mbp windows.

**Figure 4:**
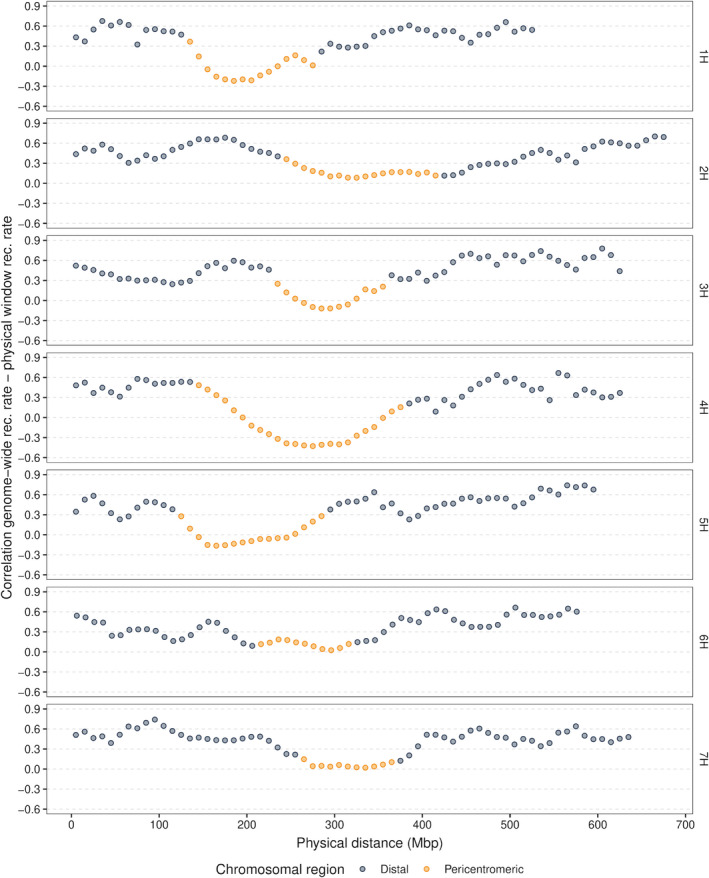
Pearson’s correlation coefficient between the 45 double round‐robin populations’ recombination rate values for 10 Mbp physical windows and their respective genome‐wide recombination rate values across the seven barley chromosomes.

The previously described genetic maps were the basis for the assessment of the recombination rates (Figures S3 and S4). The recombination rate per chromosome observed on average across all populations ranged from 0.37 (4H) to 0.58 cM/Mbp (5H) (Figure 2a). The same pattern of recombination rate along the chromosomes was also noted for the different populations (Figure 3a,b). Similar to what has been widely observed in species with large genomes such as grasses (Melamed‐Bessudo *et al*., 2016), the recombination rate was consistently found to be almost negligible in the pericentromeric region, while an increase was noticed in the distal regions. As a result, the recombination rate was found to be positively correlated with gene density (*P* < 0.001). The same trend was detected in the analysis of historical recombination in the diversity panel (Figure 3c). This was supported by the observation of a significant (*P* < 0.05) correlation coefficient between the historical and meiotic recombination rate assessed on a consensus map basis that ranged from 0.81 to 0.93. The variability of the genome‐wide recombination rate among populations was higher compared with that observed among the chromosomes, and it ranged from 0.31 to 0.73 cM/Mbp (median = 0.45 cM/Mbp). Local differences in the recombination rate were detected among populations with a median of 4.5‐fold variation in 10 Mbp windows, although some windows showed up to even a 198‐fold variation among populations (Figure 2c). The differences in the recombination rate among populations were particularly large in the distal regions, where the recombination rates varied between 1.21 and 6.45 cM/Mbp. However, when correcting for the mean differences in recombination rate across the genome by calculating the coefficient of variation, a higher recombination rate variation among populations was observed in the pericentromeric region compared with the distal regions. In addition, distal and pericentromeric regions were found distinctively correlated with the genome‐wide recombination rate (*P* < 0.001) (Figure 4). This trend was also detected when evaluating the general recombination effect (GRE) values (Figure S5).

The extent to which the above‐explained differences in recombination rates among DRR populations were due to the GRE of each of the two parental inbreds compared to the specific recombination effect (SRE) of the combination of the two involved inbreds was quantified by comparing their variances. The variance of the phenotypic estimated GRE (*GRE_P_*) values calculated genome‐wide ($\sigma_{{\text{GRE}}_{P}}^{2}$) was about eight times the variance of the SREs ($\sigma_{{\text{SRE}}_{P}}^{2}$) (Table 1). The ratio between $\sigma_{{\text{GRE}}_{P}}^{2}$ and $\sigma_{{\text{SRE}}_{P}}^{2}$ was observed to be even higher for some of the chromosomes and examined windows. The proportion of recombination rates’ variation that was due to genetic differences was measured using broad‐sense heritability (*H* ^2^) and was 0.37 on a genome‐wide level. For individual chromosomes, *H* ^2^ had values between 0.30 and 0.37, which is slightly lower compared with that for the genome‐wide recombination rate variation. Regardless of the analysed scale level, the inbreds with the largest *GRE_P_* were ItuNative, CM67, Ancap2, Lakhan, and HOR12830 (Figure 2b).

**Table 1:** The variances for the phenotypic estimated general ($\sigma_{{\text{GRE}}_{P}}^{2}$) and specific ($\sigma_{{\text{SRE}}_{P}}^{2}$) recombination effects as well as the phenotypic estimated broad‐sense heritability (*H* ^2^) for recombination rate variation.

Molecular level	$\sigma_{{\text{GRE}}_{P}}^{2}$	$\sigma_{{\text{SRE}}_{P}}^{2}$	*H* ^2^
Genome‐wide	0.00229	0.00028	0.37
Chromosomes			
1H	0.00222	0.00104	0.32
2H	0.00174	0.00107	0.30
3H	0.00302	0.00116	0.32
4H	0.00193	0.00014	0.38
5H	0.00544	0.00048	0.37
6H	0.00238	0.00040	0.36
7H	0.00223	0.00031	0.36
Windows			
Min	0.00000069	0	0.21
Max	0.17	0.14	0.63

The potential reasons for the considerable differences among the *GRE_P_* values were examined. The *GRE_P_* values of the parental inbreds were found to be significantly (*P* < 0.001) and positively (0.68) correlated with the average temperature of the geographical area where they originated from. The inbreds with the highest recombination rates originated from regions with high mean temperatures, and they were mostly six‐row types (*P* < 0.05). In contrast, annual precipitation and germplasm type were not found to be significantly correlated with *GRE_P_*. Additionally, the sequence divergence between the parental inbreds was evaluated as a factor contributing to the recombination rate variation. No significant correlation was observed between the recombination rate and allelic parental similarity neither on a population basis averaged across the genome (*P* = 0.423) nor on a genome basis averaged across the populations (*P* = 0.510; Figure S6). Across the pericentromeric region, such correlation was not significant (*P* = 0.06) on a population basis, while on a genome basis, a high negative correlation (*P* < 0.001) was observed.

### QTL analysis of CO counts

The genome‐wide CO counts ranged from 7 to 59 per DRR population with a median of 20 COs (Figure 2d). Pearson’s correlation coefficient between the average CO count per population and their recombination rates was with 0.52 highly significant (*P* < 0.001). The 1.44‐fold variation found between the populations with the lowest and highest CO count is consistent with the respective 1.35‐fold variation found for the recombination rate. On a chromosomal level, the CO counts ranged from 0 to 14 with a median between 2 and 3 COs depending on the chromosome. COs per chromosome were noted to be significantly (*P* < 0.001) correlated with the chromosomes’ physical length. Across the 8 examined CO counts, 16 quantitative trait loci (QTLs) were detected using a multi‐population analysis (Table S1). Although each detected QTL was significant (*P* < 0.05) in at least five populations (Figures S7–S10), it explained <3% of the total phenotypic variance.

### The genomic prediction of recombination‐related estimates

**Figure 5:**
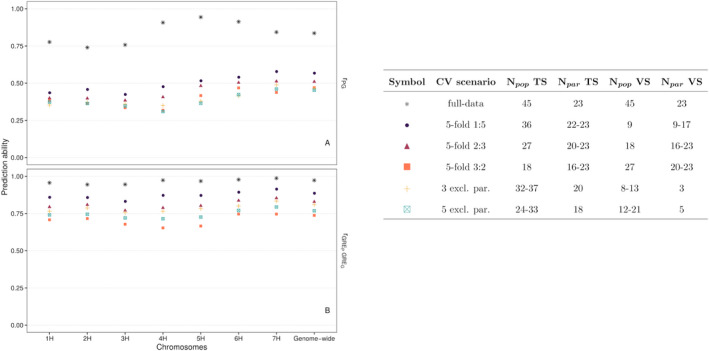
Genomic prediction ability concerning the recombination rate variation of individual chromosomes and the genome‐wide level, using different approaches. (a) Pearson’s correlation coefficient between the observed recombination rate and genomic estimated breeding values of the double round‐robin (DRR) populations, r*_PG_*. (b) Pearson’s correlation coefficient between the phenotypic and genomic estimated general recombination effects of the parental inbreds, $r_{{\text{GRE}}_{P}{\text{GRE}}_{G}}$. Cross‐validation (CV) scenarios for genomic prediction are detailed in the table, where the number of populations per validation set (*N*_pop_ VS), the number of parents involved in the populations in each validation set (*N*_par_ VS), the number of populations per training set (*N*_pop_ TS), and the number of parents involved in the populations in each training set (*N*_par_ TS) are given.

The ability to predict recombination‐related estimates for individual populations using the genome‐wide SNP profiles (Figure S11) of the parental inbreds was assessed. When using all DRR populations as the training set, the genomic best linear unbiased prediction (GBLUP) model resulted in high prediction abilities concerning the recombination rate with values between 0.74 and 0.94 for the entire genome and the individual chromosomes (Figure 5a). Cross‐validation (CV) approaches were utilized to obtain unbiased prediction abilities. The fivefold CV approach led to prediction abilities with values between 0.40 and 0.53, about 40% lower than that observed without CV. A further reduction in the training set (TS) size from 36 to 27 or even 18 lessened the ability to predict genome‐wide recombination rate by only 0.03 and 0.11, respectively, but it was slightly more pronounced for the recombination rate of the individual chromosomes. A second CV approach was implemented to test the model’s ability to predict the recombination rate for populations that were unrelated by pedigree with the populations of the TS. The prediction ability observed in these two scenarios was about 0.1 lower than those in the first CV approach with a comparable TS size.

**Figure 6:**
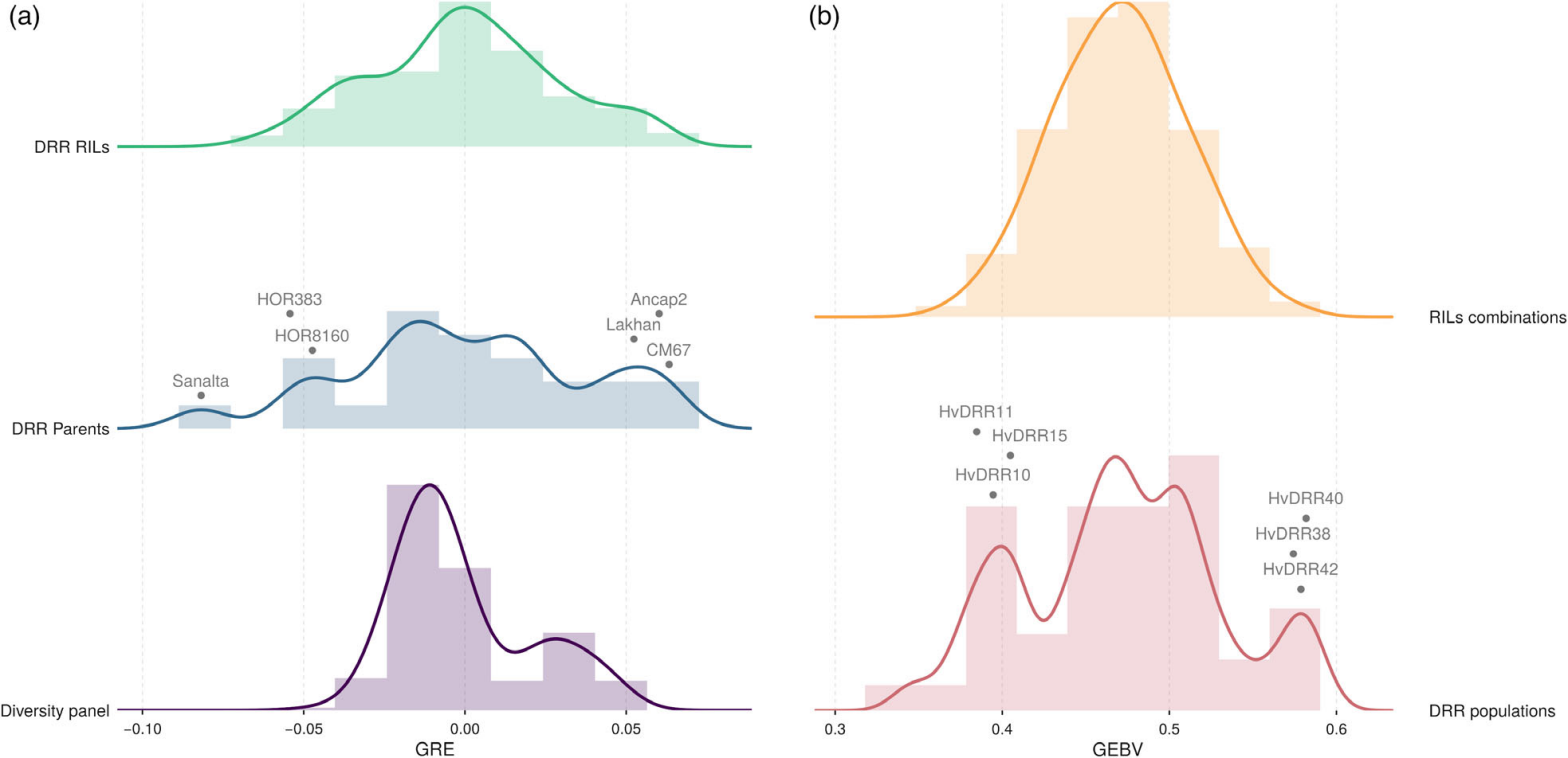
(a) Frequency distribution of the genome‐wide general recombination effects (*GRE_G_*) of the diversity panel, double round‐robin (DRR) populations’ parental lines, and DRR recombinant inbred lines (RILs) predicted by GBLUP. The *GRE_G_* for the parental inbreds of the three DRR populations with the respective lowest and highest recombination rate is displayed. (b) Frequency distribution of the genomic estimated breeding values (GEBVs) concerning the genome‐wide recombination rate for the DRR populations and for all possible populations derived from the DRR RILs. The GEBVs for the three DRR populations with the lowest and highest genome‐wide recombination rate are displayed.

In the same way, the ability to predict the GRE of parental inbreds was evaluated. The same trends outlined previously were also valid for the predicted GRE values (Figure 5b). However, the general level of the prediction ability was 13% higher compared with that concerning the recombination rate of the populations. The aforementioned analyses were also performed for the recombination rate in 10 Mbp physical windows. The model’s ability to predict recombination across individual windows was more variable and consistently lower than for entire chromosomes or genome‐wide predictions (Figure S12). In addition, the model was utilized to predict the genomic estimated GRE (*GRE_G_*) of the 3,959 DRR recombinant inbred lines (RILs). The predicted *GRE_G_* values of the RILs and the resulting genomic estimated breeding value (GEBV) of all 7,838,820 possible RILs’ hybrid combinations were observed to represent the recombination variation in parental inbreds and the DRR populations respectively (Figures 6a,b).

The ability of the model to predict the recombination of individual populations decreased by 0.02, 0.03, and 0.05 on average across the scenarios when randomly sampling a uniform distribution of SNPs across the physical map with 1 SNP per 1, 5, and 10 Mbp respectively (Figure S13). The reduction to predict the GRE of parental inbreds with the three reduced marker densities was of similar size with 0.03, 0.04, and 0.05.

## Discussion

Recent advances in understanding the mechanisms and regulation of recombination opened up biotechnological possibilities to manipulate genetic recombination (Mercier *et al*., 2015). The use of genome‐editing approaches that induce DSBs or modify epigenomes at desired sites (Hayut *et al*., 2017; Underwood *et al*., 2018) and the manipulation of CO factors (Mieulet *et al*., 2018; Sarno *et al*., 2017; Tam *et al*., 2011) are increasingly applicable for achieving this goal. However, the utilization of natural variation explored in the present study remains an alternative way to the mentioned approaches to manipulate recombination.

### The intraspecific variation of recombination rate in cultivated barley

At the genome‐wide level, the observed meiotic recombination rate ranged from 0.31 to 0.73 cM/Mbp (median = 0.45 cM/Mbp) among DRR populations (Figure 2a). Populations with the highest and lowest genome‐wide recombination rates were also among the most frequent extreme recombining populations when ranking them according to the recombination rate per individual 10 Mbp window (data not shown). This indicates that the observed genome‐wide recombination rates are not due to the effect of a few windows with a particularly high or low recombination rate in a population but because of the genome‐wide tendency.

The observed level of recombination rate variation in the DRR populations was higher than that reported in populations derived from crosses between domesticated and wild barley accessions (Dreissig *et al*., 2020). Moreover, it was also higher than most observations for other plant species such as *Arabidopsis* (Salomé *et al*., 2012; Ziolkowski *et al*., 2017), maize (Bauer *et al*., 2013), and wheat (Gardiner *et al*., 2019; Jordan *et al*., 2018), but it was lower than the values observed in the animal kingdom (Booker *et al*., 2017; Chan *et al*., 2012; Fledel‐Alon *et al*., 2011; Meznar *et al*., 2010). The high recombination rate variation observed in this study is presumably due to the high extent of genetic variation among the parental inbreds, which represent most of the genetic variation of cultivated barley (Figure 1c). A previously reported nested association mapping (NAM) population that comprised a vast genetic diversity of cultivated maize was also found to show a high variation in recombination rates among populations (McMullen *et al*., 2009).

The considerable differences noticed in the genome‐ and chromosome‐wide recombination rates among the DRR populations led to the question of whether they are caused by the effect of parental inbreds (GRE) or the specific combination of two parental inbreds (SRE); being this work the first to report them. The general effect of parental inbreds on recombination was found to be about eight times higher than the specific effect of parental combinations across the different analysed scale levels. This finding suggested that the examined parental inbreds differ in the efficiency of their recombination machinery. The segregation of structural variants in the individual DRR populations, which has been described to influence the extent and distribution of recombination events (Muñoz‐Amatriaín *et al*., 2013; Rowan *et al*., 2019; Saxena *et al*., 2014), was determined in this study as part of the SRE. This is because the same SVs will not segregate in all populations in which a common parent is involved.

Because of the high importance of the GRE in relation to the SRE in determining the recombination rate variation, it was interesting to understand the causes of the variation in GRE observed among the 23 parental inbreds. The environmental conditions at the stage of meiosis have the potential to influence the recombination rate (Wang and Copenhaver, 2018). In particular, the effect of temperature on meiosis has been studied for a long time in *Drosophila* (Plough, 1917) and plants (Dowrick, 1957), revealing that the rate of formation and distribution of COs varies depending on the temperature, as recently demonstrated in *Arabidopsis* (Choi *et al*., 2013; Lloyd *et al*., 2018; Modliszewski *et al*., 2018), barley (Higgins *et al*., 2012; Phillips *et al*., 2015), and other plants (Wang and Copenhaver, 2018), with no common trends across species. In this sense, the observed GRE and SRE might be a result of the recombination plasticity of the studied inbreds interacting with the particular environmental conditions where our crossing experiment took place. These conditions might have uncovered the different temperature responses of the examined inbreds, making those detectable as recombination rate variation. However, that is unlikely because the controlled environmental conditions under which the experiment took place are standard for barley cultivation.

Nevertheless, the *GRE_P_* values of the parental inbreds were found to be significant (*P* < 0.001) and positively correlated with the average temperature of the geographic areas where they originated from. This observation was in agreement with a previous report (Dreissig *et al*., 2019). Other environmental factors were not significantly (*P* < 0.05) correlated with the observed *GRE_P_* values.

In addition to environmental factors, the importance of genetic factors in determining the recombination rate variation was explored. As proposed in previous studies, a QTL analysis using the number of CO of each RIL as the dependent variable was performed (Esch *et al*., 2007; McMullen *et al*., 2009). To identify shared controllers of recombination across the genetic diversity of cultivated barley, a multi‐population analysis was performed, and 16 QTLs were detected. Eight of the detected QTLs were located on different chromosomes than where the CO count used as phenotype was assessed. In addition, three loci were found to be responsible for genome‐wide effects. Both observations are in accordance with reports on other plant (Dreissig *et al*., 2020; Esch *et al*., 2007; Jordan *et al*., 2018; Yandeau‐Nelson *et al*., 2006; Ziolkowski *et al*., 2017) and animal species (Fledel‐Alon *et al*., 2011; Kong *et al*., 2010), suggesting the existence of trans‐acting control of recombination. This result supports the previous explanation that the high importance of the GRE relative to the SRE in determining the recombination rate variation suggests that the barley inbreds used in this study differ in the efficiency of their recombination machinery. However, each of the detected QTLs explained <3% of the phenotypic variance (Table S1). This result is in agreement with previous studies on the genetics of intraspecific recombination in maize, wherein no shared controllers of recombination have been detected (McMullen *et al*., 2009). Furthermore, it suggests that meiotic recombination has a polygenic inheritance. The same conclusion can be drawn from the observation of a rather uniform distribution of marker effects across the genome when fitting genomic prediction models to the recombination rate estimates (Figure S11).

Finally, it must be considered that, in addition to allelic variation at the single nucleotide level, epigenetics factors are also known to play a role in the recombination of the plant genome (Henderson, 2012). In particular, recombination rate is negatively associated with the level of DNA methylation and nucleosome density (Apuli *et al*., 2020; Choi *et al*., 2013; Rowan *et al*., 2019), which partly explains the suppression for recombination in the centromeric region of chromosomes in plants where the content of heterochromatin is high (Choi *et al*., 2018). However, further research is needed to understand the effect of methylation on the recombination rate variation among different genotypes.

### Breeding for recombination rate

The amount of genetic variation generated per meiosis is determined by the extent of the recombination rate (Henderson, 2012). Therefore, recombination influences the population size as well as the number of generations required to stack multiple favourable alleles in any breeding programme (Choi and Henderson, 2015). Developing genotypes that carry alleles providing high recombination will, thus, increase the gain of selection (Jordan *et al*., 2018). The present study provides an evaluation of the procedure required to perform selection for increased recombination rates.

The considerable differences observed among the recombination rate of individual DRR populations, and especially among the GRE of parental inbreds (Figures 2a,b) as well as the moderate heritabilities (Table 1), indicate the high potential for using natural variation to manipulate the rate and distribution of recombination in the genome by systematic breeding. This approach requires the evaluation of the genetic material for its recombination properties. The high relative importance of GRE compared with SRE in determining the recombination rate variation observed in this study suggests that the recombination properties of genetic materials do not need to be evaluated in crosses with several other parental genotypes but can instead be evaluated in a resource‐efficient manner by crossing them with only one other parent. However, such approaches still require the generation of segregating material from each of the genotypes of interest, which, even with today’s genotyping and sequencing techniques, is a task that demands considerable resources. As an alternative, the prediction of recombination‐related estimates based on genome‐wide SNP profiles was evaluated for the first time in the present study. The GBLUP model using genome‐wide SNP profile data has been shown to provide a high ability to predict recombination‐related estimates (e.g. GRE) as well as the observed meiotic recombination (Figure 5). The observed prediction abilities were on a similar level to those concerning agronomic and quality traits (Haile *et al*., 2018; Heffner *et al*., 2011), where genomic prediction is routinely used in many commercial breeding programmes.

To evaluate if the observed predictive abilities were determined by the fact that the SNPs used to calculate the genetic relationship matrix were mainly located in genome regions with a high recombination rate (Figure S11), resampling simulations have been performed. Only a mild reduction in the model’s predictive ability was observed when using uniformly distributed SNPs across the genome compared to the original set of SNPs (Figure S13). That illustrates that the observed high predictive abilities are not due to the overrepresentation of those regions of the genome with high recombination rates when estimating the genetic relationship of the parental inbreds.

The impact of TS size on the prediction ability of the model was assessed through cross‐validation. As expected, the prediction ability of the model significantly decreased with smaller TS sizes in both the CV approaches performed in this study (Figure 5), which is consistent with what was previously observed in genomic selection studies in animals (VanRaden *et al*., 2009) and plants (e.g. Heffner *et al*., 2011; Lorenzana and Bernardo, 2009; Technow *et al*., 2013). However, the high prediction ability obtained with TS sizes of 27 or 18 segregating populations suggests that it is still possible to accurately select genotypes for their recombination properties using datasets that are considerably smaller than the one used in our study.

An aspect that is very important for the design of breeding programmes was examined: the ability of the model to predict the recombination rate of segregating populations derived from new inbreds, i.e., inbreds for which no other segregating populations are available in the TS. For these scenarios, only about 10% lower prediction abilities were observed compared with the CV scenarios wherein related segregating populations were in the TS of similar size (Figure 5). This finding indicates that even for new inbreds, recombination properties can be predicted reasonably well. To illustrate it further, the genome‐wide *GRE_G_* of different sets of new inbreds was predicted (Figure 6a), in addition to the GEBVs of populations derived from such new inbreds (Figure 6b). The high variation among predicted GEBVs demonstrates that the proposed method makes it possible to screen and select highly recombinogenic genotypes based on their SNPs’ profiles. These are then evaluated and recombined in the next step of recurrent selection schemes for altered recombination properties. In such a breeding scheme, the genotyped individuals are used not only to quantify the recombination rate of the parental genotypes but also to start the next cycle of a breeding programme. This, however, would not be possible when using high‐throughput pollen sequencing (Dreissig *et al*., 2015; Drouaud *et al*., 2013) instead of genotyping individual plants.

When considering the standard deviation in recombination rates among populations across the genome, it might be concluded that no variation among populations exists in the pericentromeric region (Figure 3b). However, when adjusting the variation of recombination rate among populations using the mean recombination rate in a window, to consider the coefficient of variation, a higher recombination rate variation among populations in the pericentromeric region than in the distal regions was detected. This indicated that also the recombination rate variation near the centromere should be considered when selecting genotypes for recombination.

The potential impact of increasing genetic variation in the pericentromeric region on barley breeding is particularly high, as a big portion of barley’s functional genes is present in this region (Mascher *et al*., 2017; Phillips *et al*., 2015). Therefore, the ability to predict recombination properties was evaluated not only on a genome‐ or chromosome‐wide scale but also in smaller windows across the genome. The prediction abilities observed for the recombination rate in 10 Mbp windows were considerably lower compared with that on a chromosome‐ or genome‐wide level (Figure S12). However, the ability of the model to predict the GRE for individual windows was on average >0.5 after cross‐validation, which suggests that an alteration of recombination properties in individual windows is possible.

Nevertheless, very low prediction abilities were noticed in some windows, presumably because of the low extent of variation among the *GRE_P_* of the parental inbreds for these windows. The pairwise correlations among the *GRE_P_* of individual windows and the genome‐wide *GRE_P_* were calculated to investigate if the inbreds with high genome‐wide *GRE_P_* were also those that cause a high recombination rate in the pericentromeric regions of their progenies. In this analysis, strong positive correlations were detected in the distal regions of all chromosomes, whereas most correlations in the pericentromeric region were considerably lower (Figure S5). This suggests that the mechanisms influencing recombination rate variation in distal regions differ from those in the pericentromeric region of barley chromosomes, as demonstrated in *Arabidopsis* (Choi *et al*., 2018; Rowan *et al*., 2019) and rice (Marand *et al*., 2019).

One aspect that could prevent breeders from employing our proposed procedure is if the increased recombination rate variation is negatively correlated with other important agronomic characters. However, such correlations were not observed for the barley inbreds used in this study (Table S2).

## Conclusion

The present study revealed a considerable recombination rate variation among segregating populations of the model species barley. In addition, this variation was observed to be mainly due to the general effects of individual parental inbreds, and only to about 12% of the variance was caused by combinations of both parents. Furthermore, we suggested a route and characterized the required methods to systematically manipulate recombination rates by using natural variation that might serve as an alternative or complement to controlled recombination via transgenesis.

## Experimental procedures

### Plant materials and genotypic characterization

In this work, two different genetic materials were analysed. On the one hand, 23 inbreds were selected from a diversity panel as parental inbreds of the DRR population. The inbreds were selected based on the maximal combined genotypic and phenotypic richness index (Weisweiler *et al*., 2019). The parental inbreds were then crossed following the DRR design (Stich, 2009) to initiate biparental populations (Figure 1a). Within each of the 45 populations, randomly chosen genotypes in the F2 generation underwent subsequent selfing generations to produce 35–146 RILs per population. This resulted in a total set of 3,959 RILs across 45 biparental populations, hereafter referred to as DRR populations. The cultivation of the parental inbreds to make the crosses for the F1 generation and the subsequent selfing generations, until S4 of each RIL, were carried out under identical environmental conditions in a greenhouse.

On the other hand, the diversity panel of 224 unrelated inbreds from which the parental inbreds were selected was analysed. It mostly consisted of landraces and improved varieties, representing the worldwide genetic diversity of cultivated spring barley (Haseneyer *et al*., 2009). In addition, the inbred Morex, three *ssp*. *spontaneum* (HID 4, HID 64, and HID 369), and one *ssp*. *agriocrithon* (HID 382) accessions were added to the analysis.

The RILs of the DRR populations were genotyped at the S4 generation as the occurrence of recombination events after this generation can hardly be detected because of the high degree of homozygosity. Both the RILs of the DRR populations and the inbreds from the diversity panel were genotyped by TraitGenetics GmbH (Gatersleben, Germany) using the 50K Illumina Infinium iSelect SNP genotyping array that includes 40,040 SNP markers (Bayer *et al*., 2017).

### Statistical analysis

#### Characterization of the recombination rate in segregating populations

##### Estimation of the meiotic recombination rate

The details of the data cleaning process and the procedure employed to construct the DRR populations’ linkage maps are provided in the supporting information section (Methods S1 and S2). The positions of the SNPs on the genetic maps were used together with their positions on the reference physical map (Monat *et al*., 2019) to establish a Marey map (Chakravarti, 1991) for each chromosome–DRR population combination. SNPs that did not generate a monotonously increasing trend were removed from the map but those with 2 cM diversions were tolerated (Bauer *et al*., 2013). Afterwards, a cubic spline model was fit to the coordinates of the Marey map for each chromosome–DRR population combination. Model parameters were subsequently adjusted to smooth the curve when needed (Berloff, 2002; Perperoglou *et al*., 2019). The meiotic recombination rate (*c*, cM/Mbp) (Falconer and Mackay, 1996; Petit *et al*., 2017) was calculated as the slope of the fitted curve on a 10 Mbp window basis. In case a Marey map had no SNPs in any of the extreme windows of the respective chromosome, the recombination rate for that window was estimated by deriving the predicted curve value of the average position of the five nearest SNPs.

The average recombination rate for each chromosome was calculated as the average of the recombination rates in the 10 Mbp windows of that particular chromosome. The genome‐wide recombination rate for a given population was calculated as the average recombination rate across the seven chromosomes.

The pairwise genetic similarity between both parental inbreds of each population was calculated as the fraction of shared SNP alleles on a 10 Mbp window basis. The parental similarity was correlated with the recombination rate on a genome basis (i.e. the population‐based recombination rates were averaged per physical window) as well as a population basis (i.e., the window‐based recombination rates were averaged per population). The recombination rate per physical window was correlated with the gene density in each respective window using the gene annotation provided by Monat *et al*. (2019).

##### Consensus map

A consensus map based on the 45 linkage maps of the DRR populations was developed based on the following approach: First, the average recombination rate per window across all 45 DRR populations was calculated. Then, the physical distances in Mbp between the adjacent markers (Monat *et al*., 2019) in each window were converted into cM according to the average recombination rate for that particular window. In our study, the pericentromeric region of each chromosome was defined as the continuous region surrounding the centromere for which the average recombination rate across the 45 DRR populations was 20‐fold lower than the average across the barley genome (cf. Baker *et al*., 2014). Since the pericentromeric region mostly represent the proximal region in this species, the regions of the chromosome which do not belong to the pericentromeric region were designated distal regions.

#### Calculation of historical recombination and comparison with the meiotic recombination

Historical recombination rates (ρ) were estimated using PHASE version 2.1 (Li and Stephens, 2003) for the diversity panel of 224 inbreds. To allow that the effective population size *N*_e_ can vary along the genome, the estimation of the historical recombination rate (ρ = 4*N*_e_ *c*) was performed in 2.5 Mbp windows, with an overlap of 200 Kb with neighbouring windows to avoid border effect. PHASE was evaluated with different priors of the mean historical recombination parameters (μ = 0.000002, 0.00001, 0.00002, and 0.001). Because of the observed high correlation coefficient among ρ, PHASE was finally used with the default parameter settings. The number of main iterations was increased to obtain 1,000 posterior samples (‐X10), as recommended by the authors for more accurate recombination estimates. In addition, all individuals were used in the estimations to obtain the posterior distribution for the historical recombination rate for each window (${\hat{\rho }}_{w}$). ρ_w_ was set to NA for windows with <2 variants for the diversity panel. The median of the 1,000 posterior samples of ρ_w_ was used as the point estimate (${\hat{\rho }}_{w}$).

To compare the patterns of meiotic (*c*) and historical recombination (ρ) across the barley genome, Spearman’s correlation coefficient between the average meiotic recombination rate (*c*) across the 45 DRR populations and ${\hat{\rho }}_{w}$ of the diversity panel was assessed across the 10 Mbp physical windows.

#### QTL analysis of crossover counts

The number of COs for each chromosome of each RIL as well as the sum of genome‐wide COs was the basis for this analysis. To ensure genotypic data’s quality, SNPs with a GenTrain score lower than 0.7 were excluded. In each DRR population, SNPs with missing data >10% were also discarded. In addition, RILs with >10% residual heterozygosity or missing data were discarded from each population. The CO count of each RIL was estimated using the function ‘countXO’ of the ‘R/qtl’ package (Broman *et al*., 2003). Any RIL with a CO count that exceeded by 2 COs, the last consecutive bin of its population’s frequency distribution was considered as an outlier and excluded from the analysis. A multi‐population QTL analysis was conducted using the R package ‘mppR’ (Garin *et al*., 2015), and a cross‐specific QTL effect model was considered. The significance threshold above which a position can be selected as QTL was determined as the 0.95 quantile of the null distribution created by performing 1,000 permutations.

#### Genomic prediction of the recombination rate

##### Genetic estimates of the recombination rate

The average recombination effect of a parental inbred in a series of different populations was defined as the general recombination effect (GRE), and the recombination effect of a particular population adjusted for the GRE of both involved parental inbreds was defined as the specific recombination effect (SRE). In this sense, the recombination rate *c_ij_* in a population created by crossing the parental inbreds *i* and *j* was modelled as:

(1) $$c_{\mathrm{ij}}=\mu +{\text{GRE}}_{i}+{\text{GRE}}_{j}+{\text{SRE}}_{\mathrm{ij}},$$

where μ is the intercept, *GRE* *_i_* and *GRE* *_j_* are the GRE effects of the *i* ^th^ and *j* ^th^ parental inbred, respectively, and ${\text{SRE}}_{\mathrm{ij}}$ is the SRE effect of the population between parental inbred *i* and *j*.

In the present study, two ways to estimate the GRE were evaluated: the genomically estimated GRE (*GRE* *_*G*_*) and phenotypic estimated GRE (*GRE_P_*) whose estimation procedure is described below. When the text refers to GRE without specifying whether it is genomic or phenotypic estimated, it refers to both. The estimation of SRE as well as the nomenclature of *SRE_G_* and *SRE_P_* were in analogy to that of GRE.

##### BLUP model

The meiotic recombination rate (*c*) was modelled using best linear unbiased prediction (BLUP)

(2) $$c=1_{n}\mu +Z_{\text{GRE}}u_{\text{GRE}}+Z_{\text{SRE}}u_{\text{SRE}}+e,$$

where **c** is the vector of the recombination rates for the 45 DRR populations; **1** *_n_* is the unit vector of length *n*, where *n* is the number of DRR populations; μ is the general mean; **u**_*GRE*_ and **u**_*SRE*_ are the vectors of GRE and SRE effects; and **e** is the vector of random residuals. **Z**_*GRE*_ and **Z**_*SRE*_ are the incidence matrices of the GRE and SRE effects, relating **c** to the additive (**A**) and dominance (**D**) genomic relationship matrices respectively. It is assumed that $u_{\text{GRE}}∼N\left(0,A\sigma_{a}^{2}\right)$, $u_{\text{SRE}}∼N\left(0,D\sigma_{d}^{2}\right)$, and $e∼N\left(0,I\sigma_{E}^{2}\right)$, where $\sigma_{a}^{2}$ is the additive genetic variance, $\sigma_{d}^{2}$ is the dominance variance, and $\sigma_{e}^{2}$ is the residual variance. For the calculation of *GRE_P_* and *SRE_P_*, **A** and **D** were identity matrices, as was **I**. The model fit and variance compound estimation based on REML were performed using the ‘sommer’ package (Covarrubias‐Pazaran, 2016).

##### GBLUP model

For the calculation of *GRE_G_* and *SRE_G_*, **A** and **D** were matrices from genome‐wide SNP markers, thus turning the BLUP into a GBLUP model. The SNP effect’s profiles were calculated using ridge regression best linear unbiased prediction (RR‐BLUP) (Meuwissen *et al*., 2001).

##### Prediction ability of the GBLUP model

The aforementioned model was tested to predict the *GRE_P_* of parental inbreds and the recombination rate of a population at three different scales: genome‐wide, individual chromosomes, and 10 Mbp physical windows. The ability of the model to predict the *GRE_P_* of parental inbreds was calculated using Pearson’s correlation coefficient between the phenotypic and the genomic estimated GRE of the parental inbreds ($r_{{\text{GRE}}_{P}{\text{GRE}}_{G}}$).

Moreover, the ability of the model to predict the recombination rate of a population was calculated using Pearson’s correlation coefficient between the DRR population’s recombination rate (*c*) and the GEBV of the DRR population’s recombination rate (*r_PG_)*. The latter was calculated using the model [1]. Differences between the correlations were tested for their significance using the approach proposed by Zou (2007). The broad‐sense heritability (*H* ^2^) for recombination rate was calculated as: $\frac{\sigma_{{\text{GRE}}_{P}}^{2}+\sigma_{{\text{SRE}}_{P}}^{2}}{\sigma_{{\text{GRE}}_{P}}^{2}+\sigma_{{\text{SRE}}_{P}}^{2}+\sigma_{e}^{2}}$

Additionally, the *GRE_G_* of each RIL of each DRR population was predicted using the model [2]. The GEBV of all possible combinations among DRRs’ RILs was calculated using the model [1].

##### Prediction ability evaluated by cross‐validation

Two different CV procedures were employed, with one comprising three scenarios and the other having two. The first approach was intended to evaluate the ability to predict the recombination rate of new segregating populations from parental inbreds for which already segregating populations are available. In this sense, a fivefold cross‐validation was performed by randomly dividing the full set of DRR populations into five disjoint subsets of equal size. For each of the five possible runs, one subset was left out to be used as the validation set (VS), whereas the other four subsets were used as the training set (TS). This procedure was repeated 100 times resulting in 500 cross‐validation runs in total. In addition, scenarios with different TS sizes (*N*_pop_) were evaluated by reducing the number of subsets in the TS from four (*N*_pop_ = 36) to three (27) and two (18).

The second CV approach focussed on the ability of the model to predict the GRE of new inbreds for which no segregating populations are available yet. In this approach, all populations derived from three randomly selected inbreds (*N*_par_ = 3) were used as VS and all other populations as TS. This procedure was performed 1,000 times. This analysis was also performed for *N*_par_ = 5.

The median of Pearson’s correlation coefficients across all runs of each scenario was reported and compared using a *t*‐test. The aforementioned CV approaches were performed at three different scale levels: genome‐wide, individual chromosomes, and 10 Mbp windows across the genome.

The impact of the number and distribution of SNPs on the prediction ability of the model was evaluated by repeating the above‐described procedure but using different subsets of uniformly spaced SNPs in every CV run. Three different distributions were tested: 1 SNP per 1, 5, and 10 Mbp.

#### Correlation between the recombination estimates and the characteristics of inbreds

Pearson’s correlation coefficient between the *GRE_P_* values and the variables characterizing the inbreds (such as row type, germplasm type, and geographic origin) was calculated in addition to the environment of their locations of origin (such as annual precipitation and temperature). Information about annual precipitation and the average temperature was estimated for the region of origin of each parental inbred based on historical data (1901–2016) from the World Bank’s database (The World Bank, 2020). Furthermore, Pearson’s correlation coefficient between the *GRE_P_* values and phenotypic trait scores of the parental inbreds was calculated. Procedures employed to assess phenotypic traits in field experiments are described in the supporting information section (Method S3).

## Conflict of interest

The authors declare no conflict of interest.

## Author contributions

FC performed all analyses related to meiotic recombination, DVI performed the analyses related to historical recombination and the consensus map, and contributed phenotypic information, MW contributed to SNP data analysis, JL created the segregating populations, BS designed and coordinated the project. FC, DVI, and BS wrote the manuscript. All authors read and approved the final manuscript.

## Supporting information


**Figure S1** The segregation pattern of the parental alleles across the chromosomes of the 45 double round‐robin populations.
**Figure S2** The bins’ genetic map of the seven barley chromosomes across the double round‐robin populations.
**Figure S3** The Marey maps of the seven barley chromosomes across the double round‐robin populations.
**Figure S4** The recombination landscape of the seven barley chromosomes across the double round‐robin populations.
**Figure S5** Pearson's correlation coefficient between the GRE_P_ values of the parental inbreds for 10 Mbp physical windows and their respective genome‐wide GRE_P_ values across the seven barley chromosomes.
**Figure S6** The sequence similarity between the parental inbreds across the seven barley chromosomes of the three double round‐robin populations with the highest and lowest genomic recombination rate.
**Figure S7** The effect of the QTLs associated with the number of crossovers on chromosome 2H across the 45 double round‐robin populations.
**Figure S8** The effect of the QTLs associated with the number of crossovers on chromosome 5H across the 45 double round‐robin populations.
**Figure S9** The effect of the QTLs associated with the number of crossovers on chromosome 7H across the 45 double round‐robin populations.
**Figure S10** The effect of the QTLs associated with the number of crossovers on the genome across the 45 double round‐robin populations.
**Figure S11** The distribution of SNPs' effects predicted by RR‐BLUP across the genome.
**Figure S12** The genomic prediction ability of recombination rate in 10 Mbp window level across the genome, using different cross‐validation scenarios.
**Figure S13** Genomic prediction ability concerning the recombination rate variation of individual chromosomes and the genome‐wide level, using different approaches and subsets of equally spaced SNPs.
**Table S1** Summary table of the QTLs detected for crossovers count of chromosomes 2H, 5H, and 7H, and genome‐wide using a multi‐population analysis.
**Table S2** Pearson's correlation between the phenotypic trait mean and the GRE of the parental inbreds (*r*_TGRE_) for different agronomic traits of barley.
**Method S1** Data cleaning.
**Method S2** Linkage map construction.
**Method S3** Assessment of phenotypic traits.

## Data Availability

The genotypic data utilized in this study, as well as the genetic maps and crossover counts, are available at https://doi.org/10.5281/zenodo.5495951.

## References

[pbi13746-bib-0001] Altpeter, F. , Springer, N.M. , Bartley, L.E. , Blechl, A.E. , Brutnell, T.P. , Citovsky, V. , Conrad, L.J. *et al*. (2016) Advancing crop transformation in the era of genome editing. Plant Cell, 28, 1510–1520. 27335450 10.1105/tpc.16.00196 PMC4981132

[pbi13746-bib-0002] Apuli, R.P. , Bernhardsson, C. , Schiffthaler, B. , Robinson, K.M. , Jansson, S. , Street, N.R. and Ingvarsson, P.K. (2020) ‘Inferring the genomic landscape of recombination rate variation in European Aspen (*Populus tremula*). G3: Genes ‐ Genomes ‐ Genetics, 10, 299–309. 31744900 10.1534/g3.119.400504 PMC6945010

[pbi13746-bib-0003] Arrieta, M. , Willems, G. , DePessemier, J. , Colas, I. , Burkholz, A. , Darracq, A. , Vanstraelen, S. *et al*. (2020) The effect of heat stress on sugar beet recombination. Theor. Appl. Genet. 13, 81–93. 10.1007/s00122-020-03683-0 PMC7813734 32990769

[pbi13746-bib-0004] Baker, K. , Bayer, M. , Cook, N. , Dreißig, S. , Dhillon, T. , Russell, J. , Hedley, P.E. *et al*. (2014) The low‐recombining pericentromeric region of barley restricts gene diversity and evolution but not gene expression. Plant J. 79, 981–992. 24947331 10.1111/tpj.12600 PMC4309411

[pbi13746-bib-0005] Bauer, E. , Falque, M. , Walter, H. , Bauland, C. , Camisan, C. , Campo, L. , Meyer, N. *et al*. (2013) Intraspecific variation of recombination rate in maize. Genome Biol. 14, R103. 24050704 10.1186/gb-2013-14-9-r103 PMC4053771

[pbi13746-bib-0006] Bayer, M.M. , Rapazote‐Flores, P. , Ganal, M. , Hedley, P.E. , Macaulay, M. , Plieske, J. , Ramsay, L. *et al*. (2017) Development and evaluation of a barley 50k iSelect SNP array. Front. Plant Sci. 8, 1792. 29089957 10.3389/fpls.2017.01792 PMC5651081

[pbi13746-bib-0007] Berloff (2002) Physical and genetic maps. J. Comput. Biol. 9, 465–475. 12162886 10.1089/106652702760138565

[pbi13746-bib-0008] Beyer, P. , Morell, M. , Mackay, I. and Powell, W. (2008) From mutations to MAGIC: resources for gene discovery, validation and delivery in crop plants. Curr. Opin. Plant Biol. 11, 215–221. 18295532 10.1016/j.pbi.2008.01.002

[pbi13746-bib-0009] Booker, T.R. , Ness, R.W. and Keightley, P.D. (2017) The recombination landscape in wild house mice inferred using population genomic data. Genetics, 207, 297–309. 28751421 10.1534/genetics.117.300063 PMC5586380

[pbi13746-bib-0010] Broman, K.W. , Wu, H. , Sen, S. and Churchill, G.A. (2003) R/qtl: QTL mapping in experimental crosses. Bioinformatics, 19, 889–890. 12724300 10.1093/bioinformatics/btg112

[pbi13746-bib-0011] Cederberg, H. (1985) Recombination in other chromosomal regions than the interval subjected to selection, in lines of *Neurospora crassa* selected for high and for low recombination frequency. Hereditas, 103, 89–97.

[pbi13746-bib-0012] Chakravarti, A. (1991) A graphical representation of genetic and physical maps: the Marey map. Genomics, 11, 219–222. 1765381 10.1016/0888-7543(91)90123-v

[pbi13746-bib-0013] Chan, A.H. , Jenkins, P.A. and Song, Y.S. (2012) Genome‐wide fine‐scale recombination rate variation in *Drosophila melanogaster* . PLoS Genet. 8, e1003090. 23284288 10.1371/journal.pgen.1003090 PMC3527307

[pbi13746-bib-0014] Choi, K. and Henderson, I.R. (2015) Meiotic recombination hotspots – a comparative view. Plant J. 83, 52–61. 25925869 10.1111/tpj.12870

[pbi13746-bib-0015] Choi, K. , Zhao, X. , Kelly, K.A. , Venn, O. , Higgins, J.D. , Yelina, N.E. , Hardcastle, T.J. *et al*. (2013) Arabidopsis meiotic crossover hot spots overlap with H2A.Z nucleosomes at gene promoters. Nat. Genet. 45, 1327–1338. 24056716 10.1038/ng.2766 PMC3812125

[pbi13746-bib-0016] Choi, K. , Zhao, X. , Tock, A.J. , Lambing, C. , Underwood, C.J. , Hardcastle, T.J. , Serra, H. *et al*. (2018) Nucleosomes and DNA methylation shape meiotic DSB frequency in Arabidopsis thaliana transposons and gene regulatory regions. Genome Res. 28, 532–546. 29530928 10.1101/gr.225599.117 PMC5880243

[pbi13746-bib-0017] Coop, G. , Wen, X. , Ober, C. , Pritchard, J.K. and Przeworski, M. (2008) High‐resolution mapping of crossovers reveals extensive variation in fine‐scale recombination patterns among humans. Science, 319, 1395–1398. 18239090 10.1126/science.1151851

[pbi13746-bib-0018] Covarrubias‐Pazaran, G. (2016) Genome‐assisted prediction of quantitative traits using the R package sommer. PLoS One, 11, e0156744. 27271781 10.1371/journal.pone.0156744 PMC4894563

[pbi13746-bib-0019] Darrier, B. , Rimbert, H. , Balfourier, F. , Pingault, L. , Josselin, A.A. , Servin, B. , Navarro, J. *et al*. (2017) High‐resolution mapping of crossover events in the hexaploid wheat genome suggests a universal recombination mechanism. Genetics, 206, 1373–1388. 28533438 10.1534/genetics.116.196014 PMC5500137

[pbi13746-bib-0020] Dowrick, G.J. (1957) The influence of temperature on meiosis. Heredity. 11, 37–49.

[pbi13746-bib-0021] Dreissig, S. , Fuchs, J. , Cápal, P. , Kettles, N. , Byrne, E. and Houben, A. (2015) Measuring meiotic crossovers via multi‐locus genotyping of single pollen grains in barley. PLoS One, 10, 1–10. 10.1371/journal.pone.0137677 PMC4565660 26356084

[pbi13746-bib-0022] Dreissig, S. , Mascher, M. , Heckmann, S. and Purugganan, M. (2019) Variation in recombination rate is shaped by domestication and environmental conditions in barley. Mol. Biol. Evol. 36, 2029–2039. 31209472 10.1093/molbev/msz141 PMC6736446

[pbi13746-bib-0023] Dreissig, S. , Maurer, A. , Sharma, R. , Milne, L. , Flavell, A.J. , Schmutzer, T. and Pillen, K. (2020) Natural variation in meiotic recombination rate shapes introgression patterns in intraspecific hybrids between wild and domesticated barley. New Phytol. 228, 1852–1863. 32659029 10.1111/nph.16810

[pbi13746-bib-0024] Drouaud, J. , Khademian, H. , Giraut, L. , Zanni, V. , Bellalou, S. , Henderson, I.R. , Falque, M. *et al*. (2013) Contrasted patterns of crossover and non‐crossover at *Arabidopsis thaliana* meiotic recombination hotspots. PLoS Genet. 9, e1003922. 24244190 10.1371/journal.pgen.1003922 PMC3828143

[pbi13746-bib-0025] Dumont, B.L. , Broman, K.W. and Payseur, B.A. (2009) Variation in genomic recombination rates among heterogeneous stock mice. Genetics, 182, 1345–1349. 19535547 10.1534/genetics.109.105114 PMC2728871

[pbi13746-bib-0026] Esch, E. , Szymaniak, J.M. , Yates, H. , Pawlowski, W.P. and Buckler, E.S. (2007) Using crossover breakpoints in recombinant inbred lines to identify quantitative trait loci controlling the global recombination frequency. Genetics, 177, 1851–1858. 17947409 10.1534/genetics.107.080622 PMC2147985

[pbi13746-bib-0027] Falconer, D.S. and Mackay, T.F.C. (1996) Introduction to Quantitative Genetics, 4th ed. Essex, UK: Longman.

[pbi13746-bib-0028] Fledel‐Alon, A. , Leffler, E.M. , Guan, Y. , Stephens, M. , Coop, G. and Przeworski, M. (2011) Variation in human recombination rates and its genetic determinants. PLoS One, 6, e20321. 21698098 10.1371/journal.pone.0020321 PMC3117798

[pbi13746-bib-0029] Gardiner, L.J. , Wingen, L.U. , Bailey, P. , Joynson, R. , Brabbs, T. , Wright, J. , Higgins, J.D. *et al*. (2019) Analysis of the recombination landscape of hexaploid bread wheat reveals genes controlling recombination and gene conversion frequency. Genome Biol. 20, 1. 30982471 10.1186/s13059-019-1675-6 PMC6463664

[pbi13746-bib-0030] Garin, V. , Wimmer, V. and Malosetti, M. (2015) mppR: an R package for QTL analysis in multi‐parent populations using linear mixed models. R Vignette. https://cran.r‐project.org/package=mppR/vignettes/mppR_vignette.pdf

[pbi13746-bib-0031] Gion, J.M. , Hudson, C.J. , Lesur, I. , Vaillancourt, R.E. , Potts, B.M. and Freeman, J.S. (2016) Genome‐wide variation in recombination rate in Eucalyptus. BMC Genom. 17, 1–12. 10.1186/s12864-016-2884-y PMC4979139 27507140

[pbi13746-bib-0032] Haile, J.K. , N’Diaye, A. , Clarke, F. , Clarke, J. , Knox, R. , Rutkoski, J. , Bassi, F.M. *et al*. (2018) Genomic selection for grain yield and quality traits in durum wheat. Mol. Breed. 38, 75.

[pbi13746-bib-0033] Haseneyer, G. , Stracke, S. , Paul, C. , Einfeldt, C. , Broda, A. , Piepho, H.‐P. , Graner A. *et al*. (2009) Population structure and phenotypic variation of a spring barley world collection set up for association studies. Plant Breed. 129, 271–279.

[pbi13746-bib-0034] Hayta, S. , Smedley, M.A. , Demir, S.U. , Blundell, R. , Hinchliffe, A. , Atkinson, N. and Harwood, W.A. (2019) An efficient and reproducible *Agrobacterium*‐mediated transformation method for hexaploid wheat (*Triticum aestivum* L.). Plant Methods, 15, 1–15. 31673278 10.1186/s13007-019-0503-z PMC6815027

[pbi13746-bib-0035] Hayut, S.F. , Bessudo, C.M. and Levy, A.A. (2017) Targeted recombination between homologous chromosomes for precise breeding in tomato. Nat. Commun. 8, 15605. 28548094 10.1038/ncomms15605 PMC5458649

[pbi13746-bib-0036] Heffner, E.L. , Jannink, J.L. , Iwata, H. , Souza, E. and Sorrells, M.E. (2011) Genomic selection accuracy for grain quality traits in biparental wheat populations. Crop Sci. 51, 2597–2606.

[pbi13746-bib-0037] Henderson, I.R. (2012) Control of meiotic recombination frequency in plant genomes. Curr. Opin. Plant Biol. 15, 556–561. 23017241 10.1016/j.pbi.2012.09.002

[pbi13746-bib-0038] Higgins, J.D. , Perry, R.M. , Barakate, A. , Ramsay, L. , Waugh, R. , Halpin, C. , Armstrong, S.J. *et al*. (2012) Spatiotemporal asymmetry of the meiotic program underlies the predominantly distal distribution of meiotic crossovers in barley. Plant Cell, 24, 4096–4109. 23104831 10.1105/tpc.112.102483 PMC3517238

[pbi13746-bib-0039] Hunter, C.M. , Huang, W. , Mackay, T.F. and Singh, N.D. (2016) The genetic architecture of natural variation in recombination rate in *Drosophila melanogaster* . PLoS Genet. 12, 1–31. 10.1371/journal.pgen.1005951 PMC4817973 27035832

[pbi13746-bib-0040] Jordan, K.W. , Wang, S. , He, F. , Chao, S. , Lun, Y. , Paux, E. , Sourdille, P. *et al*. (2018) The genetic architecture of genome‐wide recombination rate variation in allopolyploid wheat revealed by nested association mapping. Plant J. 95, 1039–1054. 29952048 10.1111/tpj.14009 PMC6174997

[pbi13746-bib-0041] Kim, S. , Plagnol, V. , Hu, T.T. , Toomajian, C. , Clark, R.M. , Ossowski, S. , Ecker, J.R. *et al*. (2007) Recombination and linkage disequilibrium in *Arabidopsis thaliana* . Nat. Genet. 39, 1151–1155. 17676040 10.1038/ng2115

[pbi13746-bib-0042] Kong, A. , Thorleifsson, G. , Gudbjartsson, D.F. , Masson, G. , Sigurdsson, A. , Jonasdottir, A.A. , Walters, G.B. *et al*. (2010) Fine‐scale recombination rate differences between sexes, populations and individuals. Nature, 467, 1099–1103. 20981099 10.1038/nature09525

[pbi13746-bib-0043] Lee, M. , Sharopova, N. , Beavis, W.D. , Grant, D. , Katt, M. , Blair, D. and Hallauer, A. (2002) Expanding the genetic map of maize with the intermated B73 x Mo17 (IBM) population. Plant Mol. Biol. 48, 453–461. 11999829 10.1023/a:1014893521186

[pbi13746-bib-0044] Li, N. and Stephens, M. (2003) Modeling linkage disequilibrium and identifying recombination hotspots using single‐nucleotide polymorphism data. Genetics, 165, 2213–2233. 14704198 10.1093/genetics/165.4.2213 PMC1462870

[pbi13746-bib-0045] Lloyd, A. , Morgan, C. , Franklin, F.C.H. and Bomblies, K. (2018) Plasticity of meiotic recombination rates in response to temperature in arabidopsis. Genetics, 208, 1409–1420. 29496746 10.1534/genetics.117.300588 PMC5887139

[pbi13746-bib-0046] Lorenzana, R.E. and Bernardo, R. (2009) Accuracy of genotypic value predictions for marker‐based selection in biparental plant populations. Theor. Appl. Genet. 120, 151–161. 19841887 10.1007/s00122-009-1166-3

[pbi13746-bib-0047] Marand, A.P. , Zhao, H. , Zhang, W. , Zeng, Z. , Fang, C. and Jianga, J. (2019) Historical meiotic crossover hotspots fueled patterns of evolutionary divergence in rice. Plant Cell, 31, 645–662. 30705136 10.1105/tpc.18.00750 PMC6482639

[pbi13746-bib-0048] Mascher, M. , Gundlach, H. , Himmelbach, A. , Beier, S. , Twardziok, S.O. , Wicker, T. , Radchuk, V. *et al*. (2017) A chromosome conformation capture ordered sequence of the barley genome. Nature, 544, 427–433. 28447635 10.1038/nature22043

[pbi13746-bib-0049] McMullen, M.D. , Kresovich, S. , Villeda, H.S. , Bradbury, P. , Li, H. , Sun, Q. , Flint‐Garcia, S. *et al*. (2009) Genetic properties of the maize nested association mapping population. Science, 325, 737–740. 19661427 10.1126/science.1174320

[pbi13746-bib-0050] Melamed‐Bessudo, C. , Shilo, S. and Levy, A.A. (2016) Meiotic recombination and genome evolution in plants. Curr. Opin. Plant Biol. 30, 82–87. 26939088 10.1016/j.pbi.2016.02.003

[pbi13746-bib-0051] Mercier, R. , Mézard, C. , Jenczewski, E. , Macaisne, N. and Grelon, M. (2015) The molecular biology of meiosis in plants. Annu. Rev. Plant Biol. 66, 297–327. 25494464 10.1146/annurev-arplant-050213-035923

[pbi13746-bib-0052] Meuwissen, T.H. , Hayes, B.J. and Goddard, M.E. (2001) Prediction of total genetic value using genome‐wide dense marker maps. Genetics, 157, 1819–1829. 11290733 10.1093/genetics/157.4.1819 PMC1461589

[pbi13746-bib-0053] Meznar, E.R. , Gadau, J. , Koeniger, N. and Rueppell, O. (2010) Comparative linkage mapping suggests a high recombination rate in all honeybees. J. Hered. 101, S118–S126. 20212006 10.1093/jhered/esq002

[pbi13746-bib-0054] Mieulet, D. , Aubert, G. , Bres, C. , Klein, A. , Droc, G. , Vieille, E. , Rond‐Coissieux, C. *et al*. (2018) Unleashing meiotic crossovers in crops. Nat. Plants, 4, 1010–1016. 30478361 10.1038/s41477-018-0311-x

[pbi13746-bib-0055] Modliszewski, J.L. , Wang, H. , Albright, A.R. , Lewis, S.M. , Bennett, A.R. , Huang, J. , Ma, H. *et al*. (2018) Elevated temperature increases meiotic crossover frequency via the interfering (Type I) pathway in Arabidopsis thaliana. PLoS Genet. 14, 1–15. 10.1371/journal.pgen.1007384 PMC5976207 29771908

[pbi13746-bib-0056] Monat, C. , Padmarasu, S. , Lux, T. , Wicker, T. , Gundlach, H. , Himmelbach, A. , Ens, J. *et al*. (2019) TRITEX: chromosome‐scale sequence assembly of Triticeae genomes with open‐source tools. Genome Biol. 20, 284. 31849336 10.1186/s13059-019-1899-5 PMC6918601

[pbi13746-bib-0057] Morgan, H. (1916) A Critique of the Theory of Evolution. Princeton, NJ: Princeton University Press.

[pbi13746-bib-0058] Muñoz‐Amatriaín, M. , Eichten, S.R. , Wicker, T. , Richmond, T.A. , Mascher, M. , Steuernagel, B. , Scholz, U. *et al*. (2013) Distribution, functional impact, and origin mechanisms of copy number variation in the barley genome. Genome Biol. 14, R58. 23758725 10.1186/gb-2013-14-6-r58 PMC3706897

[pbi13746-bib-0059] Nachman, M.W. (2002) Variation in recombination rate across the genome: evidence and implications. Curr. Opin. Genet. Dev. 12, 657–663. 12433578 10.1016/s0959-437x(02)00358-1

[pbi13746-bib-0060] Perperoglou, A. , Sauerbrei, W. , Abrahamowicz, M. and Schmid, M. (2019) A review of spline function procedures in R. BMC Med. Res. Methodol. 19, 1–16. 30841848 10.1186/s12874-019-0666-3 PMC6402144

[pbi13746-bib-0061] Petit, M. , Astruc, J.‐M. , Sarry, J. , Drouilhet, L. , Fabre, S. , Moreno, C.R. and Servin, B. (2017) Variation in recombination rate and its genetic determinism in sheep populations. Genetics, 207, 767–784. 28978774 10.1534/genetics.117.300123 PMC5629338

[pbi13746-bib-0062] Phillips, D. , Jenkins, G. , Macaulay, M. , Nibau, C. , Wnetrzak, J. , Fallding, D. , Colas, I. *et al*. (2015) The effect of temperature on the male and female recombination landscape of barley. New Phytol. 208, 421–429. 26255865 10.1111/nph.13548

[pbi13746-bib-0063] Plough, H.H. (1917) The effect of temperature on crossingover in *Drosophila* . J. Exp. Zool. 24, 147–209.

[pbi13746-bib-0064] Rodgers‐Melnick, E. , Bradbury, P.J. , Elshire, R.J. , Glaubitz, J.C. , Acharya, C.B. , Mitchell, S.E. , Li, C. *et al*. (2015) Recombination in diverse maize is stable, predictable, and associated with genetic load. Proc. Natl Acad. Sci. USA, 112, 3823–3828. 25775595 10.1073/pnas.1413864112 PMC4378432

[pbi13746-bib-0065] Rowan, B.A. , Heavens, D. , Feuerborn, T.R. , Tock, A.J. , Henderson, I.R. and Weigel, D. (2019) An ultra high‐density *Arabidopsis thaliana* crossover. Genetics, 213, 771–787. 31527048 10.1534/genetics.119.302406 PMC6827372

[pbi13746-bib-0066] Salomé, P.A. , Bomblies, K. , Fitz, J. , Laitinen, R.A. , Warthmann, N. , Yant, L. and Weigel, D. (2012) The recombination landscape in *Arabidopsis thaliana* F2 populations. Heredity (Edinb), 108, 447–455. 22072068 10.1038/hdy.2011.95 PMC3313057

[pbi13746-bib-0067] Sandor, C. , Li, W. , Coppieters, W. , Druet, T. , Charlier, C. and Georges, M. (2012) Genetic variants in REC8, RNF212, and PRDM9 influence male recombination in cattle. PLoS Genet. 8, e1002854. 22844258 10.1371/journal.pgen.1002854 PMC3406008

[pbi13746-bib-0068] Sarno, R. , Vicq, Y. , Uematsu, N. , Luka, M. , Lapierre, C. , Carroll, D. , Bastianelli, G. *et al*. (2017) Programming sites of meiotic crossovers using Spo11 fusion proteins. Nucleic Acids Res. 45, e164. 28977556 10.1093/nar/gkx739 PMC5737382

[pbi13746-bib-0069] Saxena, R.K. , Edwards, D. and Varshney, R.K. (2014) Structural variations in plant genomes. Brief. Funct. Genomics, 13, 296–307. 24907366 10.1093/bfgp/elu016 PMC4110416

[pbi13746-bib-0070] Shen, C. , Wang, N. , Huang, C. , Wang, M. , Zhang, X. and Lin, Z. (2019) Population genomics reveals a fine‐scale recombination landscape for genetic improvement of cotton. Plant J. 99, 494–505. 31002209 10.1111/tpj.14339

[pbi13746-bib-0071] Smeds, L. , Mugal, C.F. , Qvarnström, A. and Ellegren, H. (2016) High‐resolution mapping of crossover and non‐crossover recombination events by whole‐genome re‐sequencing of an avian pedigree. PLoS Genet. 12, 1–23. 10.1371/journal.pgen.1006044 PMC4878770 27219623

[pbi13746-bib-0072] Stich, B. (2009) Comparison of mating designs for establishing nested association mapping populations in maize and *Arabidopsis thaliana* . Genetics, 183, 1525–1534. 19805816 10.1534/genetics.109.108449 PMC2787436

[pbi13746-bib-0073] Taagen, E. , Bogdanove, A.J. and Sorrells, M.E. (2020) Counting on crossovers: controlled recombination for plant breeding. Trends Plant Sci. 25, 455–465. 31959421 10.1016/j.tplants.2019.12.017

[pbi13746-bib-0074] Tam, S.M. , Hays, J.B. and Chetelat, R.T. (2011) Effects of suppressing the DNA mismatch repair system on homeologous recombination in tomato. Theor. Appl. Genet. 123, 1445–1458. 21870137 10.1007/s00122-011-1679-4

[pbi13746-bib-0075] Technow, F. , Bürger, A. and Melchinger, A.E. (2013) Genomic prediction of northern corn leaf blight resistance in maize with combined or separated training sets for heterotic groups. G3: Genes ‐ Genomes ‐ Genetics, 3, 197–203. 23390596 10.1534/g3.112.004630 PMC3564980

[pbi13746-bib-0076] The World Bank (2020) World Development Indicators. Accessed June 2020. https://data.worldbank.org/.

[pbi13746-bib-0077] Tiley, G.P. and Burleigh, G. (2015) The relationship of recombination rate, genome structure, and patterns of molecular evolution across angiosperms. BMC Evol. Biol. 15, 1–14. 26377000 10.1186/s12862-015-0473-3 PMC4574184

[pbi13746-bib-0078] Underwood, C.J. , Choi, K. , Lambing, C. , Zhao, X. , Serra, H. , Borges, F. , Simorowski, J. *et al*. (2018) Epigenetic activation of meiotic recombination near Arabidopsis thaliana centromeres via loss of H3K9me2 and non‐CG DNA methylation. Genome Res. 28, 519–531. 29530927 10.1101/gr.227116.117 PMC5880242

[pbi13746-bib-0079] VanRaden, P.M. , Van Tassell, C.P. , Wiggans, G.R. , Sonstegard, T.S. , Schnabel, R.D. , Taylor, J.F. and Schenkel, F.S. (2009) Invited review: reliability of genomic predictions for north american Holstein bulls. J. Dairy Sci. 92, 16–24. 19109259 10.3168/jds.2008-1514

[pbi13746-bib-0080] Wang, Y. and Copenhaver, G.P. (2018) Meiotic recombination: mixing it up in plants. Annu. Rev. Plant Biol. 69, 577–609. 29489392 10.1146/annurev-arplant-042817-040431

[pbi13746-bib-0081] Weisweiler, M. , Montaigu, A.D. , Ries, D. , Pfeifer, M. and Stich, B. (2019) Transcriptomic and presence/absence variation in the barley genome assessed from multi‐tissue mRNA sequencing and their power to predict phenotypic traits. BMC Genom., 20, 1–15. 10.1186/s12864-019-6174-3 PMC6819542 31664921

[pbi13746-bib-0082] Weng, Z.Q. , Saatchi, M. , Schnabel, R.D. , Taylor, J.F. and Garrick, D.J. (2014) Recombination locations and rates in beef cattle assessed from parent‐offspring pairs. Genet. Sel. Evol. 46, 1–14. 10.1186/1297-9686-46-34 PMC4071795 24885305

[pbi13746-bib-0083] Yandeau‐Nelson, M.D. , Nikolau, B.J. and Schnable, P.S. (2006) Effects of trans‐acting genetic modifiers on meiotic recombination across the a1‐sh2 interval of maize. Genetics, 174, 101–112. 16816431 10.1534/genetics.105.049270 PMC1569796

[pbi13746-bib-0084] Yu, J. , Holland, J.B. , McMullen, M.D. and Buckler, E.S. (2008) Genetic design and statistical power of nested association mapping in maize. Genetics, 178, 539–551. 18202393 10.1534/genetics.107.074245 PMC2206100

[pbi13746-bib-0085] Ziolkowski, P.A. , Underwood, C.J. , Lambing, C. , Martinez‐Garcia, M. , Lawrence, E.J. , Ziolkowska, L. , Griffin, C. *et al*. (2017) Natural variation and dosage of the HEI10 meiotic E3 ligase control *Arabidopsis* crossover recombination. Genes Dev. 31, 306–317. 28223312 10.1101/gad.295501.116 PMC5358726

[pbi13746-bib-0086] Zou, G.Y. (2007) Toward using confidence intervals to compare correlations. Psychol. Methods, 12, 399–413. 18179351 10.1037/1082-989X.12.4.399

