# Supplementary material for: Genomic prediction of the recombination rate variation in barley – A route to highly recombinogenic genotypes
Source: Plant Biotechnol J. 2021 Dec 11;20(4):676–90. doi: 10.1111/pbi.13746 (PMC8989500; doi:10.1111/pbi.13746)
Supplement: Supplementary file 1 — Figure S1 The segregation pattern of the parental alleles across the chromosomes of the 45 double round‐robin populations. Figure S2 The bins’ genetic map of the seven barley chromosomes across the double round‐robin populations. Figure S3 The Marey maps of the seven barley chromosomes across the double round‐robin populations. Figure S4 The recombination landscape of the seven barley chromosomes across the double round‐robin populations. Figure S5 Pearson's correlation coefficient between the GREP values of the parental inbreds for 10 Mbp physical windows and their respective genome‐wide GREP values across the seven barley chromosomes. Figure S6 The sequence similarity between the parental inbreds across the seven barley chromosomes of the three double round‐robin populations with the highest and lowest genomic recombination rate. Figure S7 The effect of the QTLs associated with the number of crossovers on chromosome 2H across the 45 double round‐robin populations. Figure S8 The effect of the QTLs associated with the number of crossovers on chromosome 5H across the 45 double round‐robin populations. Figure S9 The effect of the QTLs associated with the number of crossovers on chromosome 7H across the 45 double round‐robin populations. Figure S10 The effect of the QTLs associated with the number of crossovers on the genome across the 45 double round‐robin populations. Figure S11 The distribution of SNPs' effects predicted by RR‐BLUP across the genome. Figure S12 The genomic prediction ability of recombination rate in 10 Mbp window level across the genome, using different cross‐validation scenarios. Figure S13 Genomic prediction ability concerning the recombination rate variation of individual chromosomes and the genome‐wide level, using different approaches and subsets of equally spaced SNPs. Table S1 Summary table of the QTLs detected for crossovers count of chromosomes 2H, 5H, and 7H, and genome‐wide using a multi‐population analysis. Table S2 Pearson's correlation [file PBI-20-676-s001.pdf]

SUPPORTING INFORMATION

Methods S1: Data cleaning

SNPs with a GenTrain score lower than 0.4 were excluded. In each DRR population, monomorphic SNPs and those with a minor allele frequency (MAF) $< 10\%$ were discarded. Further, SNPs with $> 20\%$ of missing data or exhibiting heterozygosity $> 20\%$ were eliminated from the data set. SNPs showing segregation distortion were identified with a Chi-square (X^2) Goodness-of-fit test ($P < 0.05$) after applying the Bonferroni correction for multiple tests and were excluded from map construction (Ott and Longnecker, 2006). A segregation distortion region (SDR) was considered large if it occupied a segment longer than 50 Mbp in which more than 80% of the markers were found significantly distorted. In the same way as SNPs, RILs with $> 20\%$ residual heterozygosity or missing data were discarded. RILs carrying nonparental alleles were also removed from the dataset. The resulting dataset comprised 36,077 SNPs across the total set of DRR populations with 34 to 143 RILs per population. A principal coordinate analysis was performed on the DRR populations and their parental inbreds based on the modified Roger's distance calculated from that dataset. Following the same procedure, a principal coordinate analysis of the diversity panel, Morex, three *ssp. spontaneum*, and one *ssp. agriocrithon* accessions, was performed.

In the context of assessing the historical recombination, monomorphic SNPs and SNPs with a MAF $< 5\%$ were removed from the dataset of the diversity panel. Moreover, SNPs with $> 10\%$ heterozygotes and $> 20\%$ missing data were discarded. For those individuals with $> 10\%$ heterozygote SNPs, the SNP genotypes were replaced by NA. Only SNPs with a position on the physical reference map were retained (Monat et al., 2019). This cleaning process resulted in a total of 35,980 SNPs.

Methods S2: Linkage map construction

Only one of the perfectly co-segregating SNPs (referred to as duplicated SNPs) was retained to increase computational efficiency. To start the map construction, SNPs in each DRR population were categorized to seven linkage groups using the R (R Core Team, 2020) package "qtl" (Broman et al., 2003). Then, Carthagene (de Givry et al., 2005) was used to create the genetic map of each linkage group in four steps: sketch, scaffold, framework, and addition of the remaining markers. First, a comprehensive map for each linkage group was sketched using "mfmapd" function. SNPs 10 centimorgans (cM) apart on the sketch map were selected to construct the scaffold map of the respective linkage group, using "buildfw" function. The resulting scaffold map was re-ordered with the "flips" command. In the next step, the remaining SNPs of each linkage group were added to the respective scaffold map using the "buildfw" function to construct high-density framework maps followed by using the "flips" function to improve the order. This addition of

SNPs and the ordering was repeated twice with decreasing LOD thresholds. After the framework map's construction, the only SNPs that remained unmapped were those that did not produce a change in the log-likelihood of the map after being added to the framework map. These SNPs were then added to the respective framework map at their best position. Finally, duplicated SNPs were included in their co-segregating bin to obtain the final map. The genetic distances were estimated using Haldane's mapping function (Haldane, 1919).

Methods S3: Assessment of phenotypic traits

For the assessment of phenotypic traits under field conditions, the 23 parental inbreds were planted as replicated check genotypes in an experiment with other entries which was laid out as an augmented row-column design. This experiment was repeated in seven environments in Germany: Cologne (2017, 2018, and 2019), Mechernich (2018 and 2019), and Quedlinburg (2018 and 2019). The leaf angle was assessed in four-week-old plants, the heading date was scored as the number of days after planting (DAP), and the plant height was assessed after heading. The seed traits were measured with MARVIN seed analyzer (GTA Sensorik, Neubrandenburg, Germany).

REFERENCES

Broman, K. W., Wu, H., Sen, Ś. and Churchill, G. A. (2003), 'R/qtl: QTL mapping in experimental crosses', *Bioinformatics* **19**, 889–890.

de Givry, S., Bouchez, M., Chabrier, P., Milan, D. and Schiex, T. (2005), 'CARTHAGENE: multipopulation integrated genetic and radiation hybrid mapping', *Bioinformatics* **21**, 1703–1704.

Haldane, J. B. (1919), 'The combination of linkage values, and the calculation of distances between the loci of linked factors', *J. Genet.* **8**, 299–309.

Monat, C., Padmarasu, S., Lux, T., Wicker, T., Gundlach, H., Himmelbach, A., Ens, J., Li, C., Muehlbauer, G. J., Schulman, A. H., Waugh, R., Braumann, I., Pozniak, C., Scholz, U., Mayer, K. F., Spannagl, M., Stein, N. and Mascher, M. (2019), 'TRITEX: chromosome-scale sequence assembly of Triticeae genomes with open-source tools', *Genome Biol.* **20**, 284.

Ott, R. L. and Longnecker, M. T. (2006), *Introduction to statistical methods and data analysis*, Duxbury Press, USA.

R Core Team (2020), *R: a language and environment for statistical computing*, R Foundation for Statistical Computing, Vienna, Austria.

Table S1: Summary table of the QTLs detected for crossovers count of chromosomes 2H, 5H, and 7H, and genome-wide using a multi-population analysis. R^2 is the percentage of the explained phenotypic variance. N populations is the number of double round-robin populations in which the QTL was found significant.

**QTL**	**Phenotype**	**Chromosome**	**Physical interval**	R^2	**N populations**
1	chr: 2H	2H	19,706,917 – 21,564,939	2.13	9
2	chr: 2H	3H	540,528,678 – 549,939,046	2.14	10
3	chr: 2H	4H	476,635,174 – 496,775,512	2.17	12
4	chr: 2H	5H	526,241,270 – 528,726,782	2.95	9
5	chr: 2H	6H	478,413,233 – 501,926,097	2.84	11
6	chr: 2H	7H	5,146,581 – 13,654,310	1.92	10
7	chr: 5H	2H	5,283,335 – 10,970,690	2.19	9
8	chr: 5H	5H	447,633,911 – 523,261,050	2.17	10
9	chr: 5H	5H	484,772,895 – 488,160,271	3.27	9
10	chr: 5H	5H	585,716,037 – 588,620,278	2.49	11
11	chr: 7H	4H	529,604,097 – 557,962,172	2.24	9
12	chr: 7H	5H	533,736,593 – 548,597,684	2.79	11
13	chr: 7H	7H	632,263,733 – 632,995,979	2.19	5
14	genome-wide	1H	477,085,105 – 478,327,181	2.60	7
15	genome-wide	4H	580,387,883 – 586,286,949	2.57	10
16	genome-wide	5H	516,799,912 – 519,129,769	2.69	10

Table S2: Pearson's correlation between the phenotypic trait mean and the GRE of the parental inbreds (r_{TGRE}) for different agronomic traits of barley.

**Trait**	r_{TGRE}
Leaf angle	-0.15^{ns}
Heading date	-0.31^{ns}
Plant height	-0.11^{ns}
Thousand seed weight	-0.25^{ns}
Seed length	-0.07^{ns}
Seed width	-0.24^{ns}
Seed area	-0.19^{ns}

ns, not significant

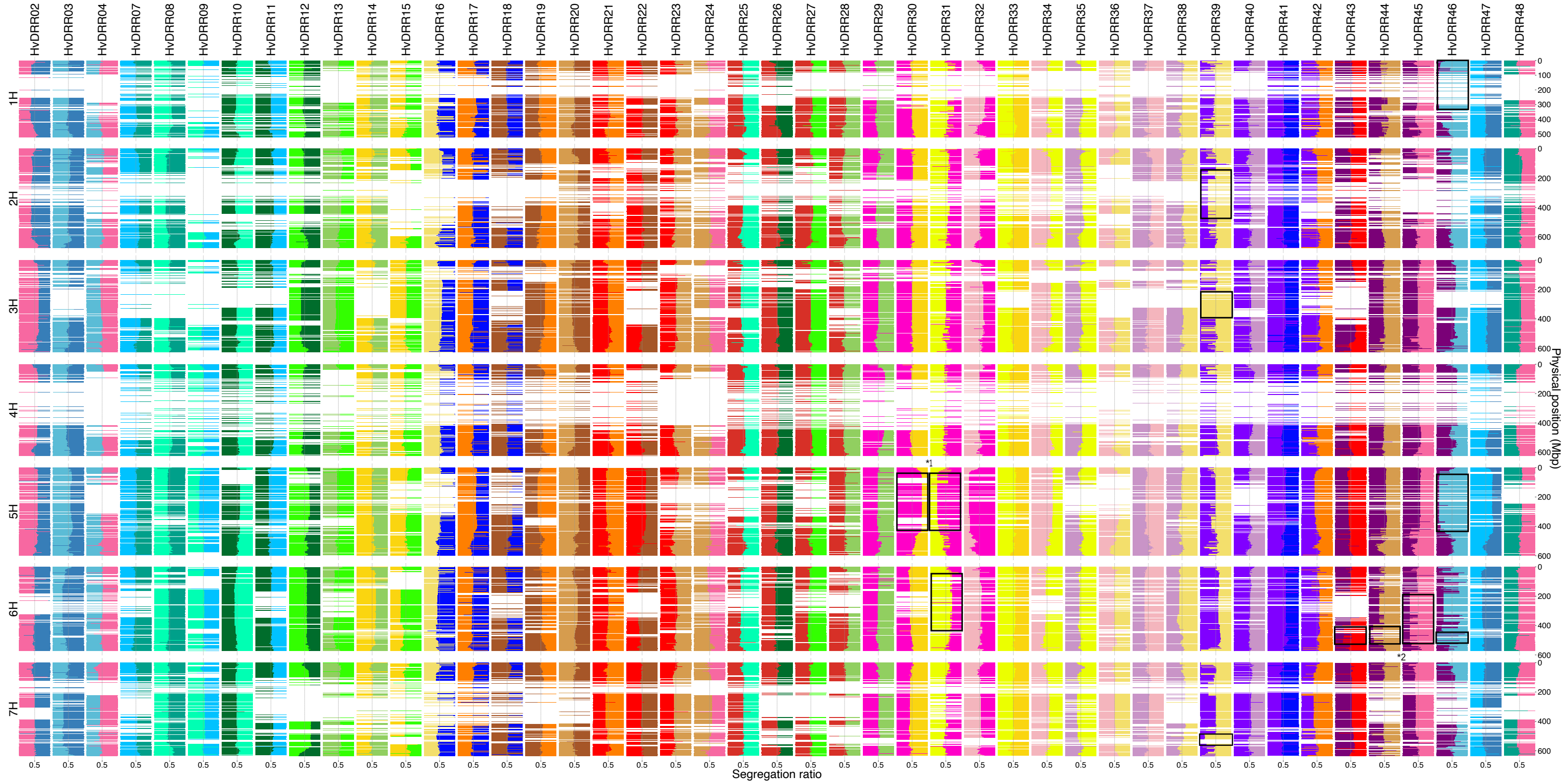

Fig. S1: The segregation pattern of the parental alleles across the chromosomes of the 45 double round-robin populations. For each SNP, the allele frequency of the two parental alleles in each population is displayed as colored horizontal bar. A particular parental inbred is represented by the same color across all populations. Large regions with significant segregation distortion (SDR) are marked as dark rectangles, where an SDR that favored a common parent is identified with *1, and an SDR that disfavored a common parent is identified with *2.

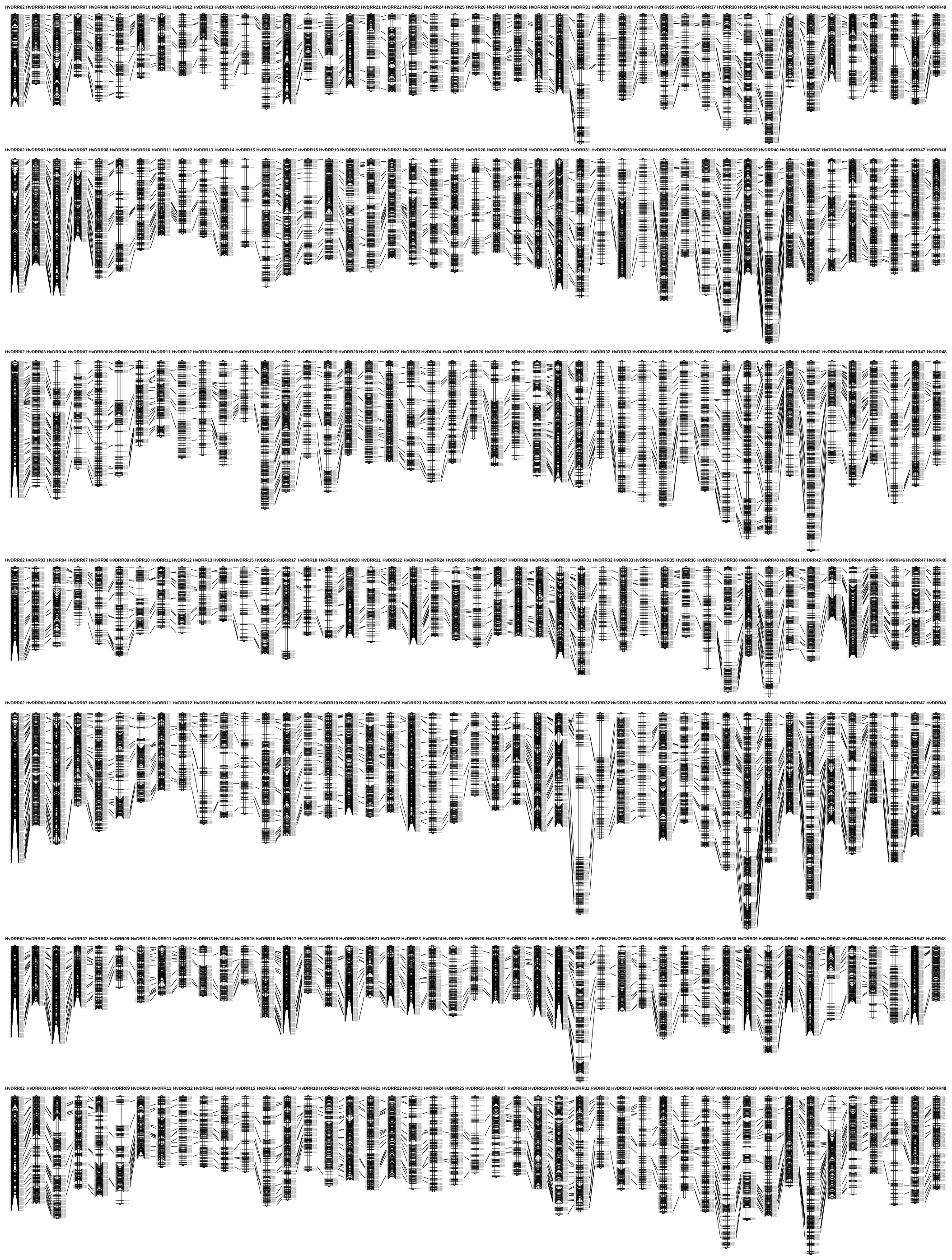

Fig. S2: The bins' genetic map of the seven barley chromosomes across the double round-robin populations.

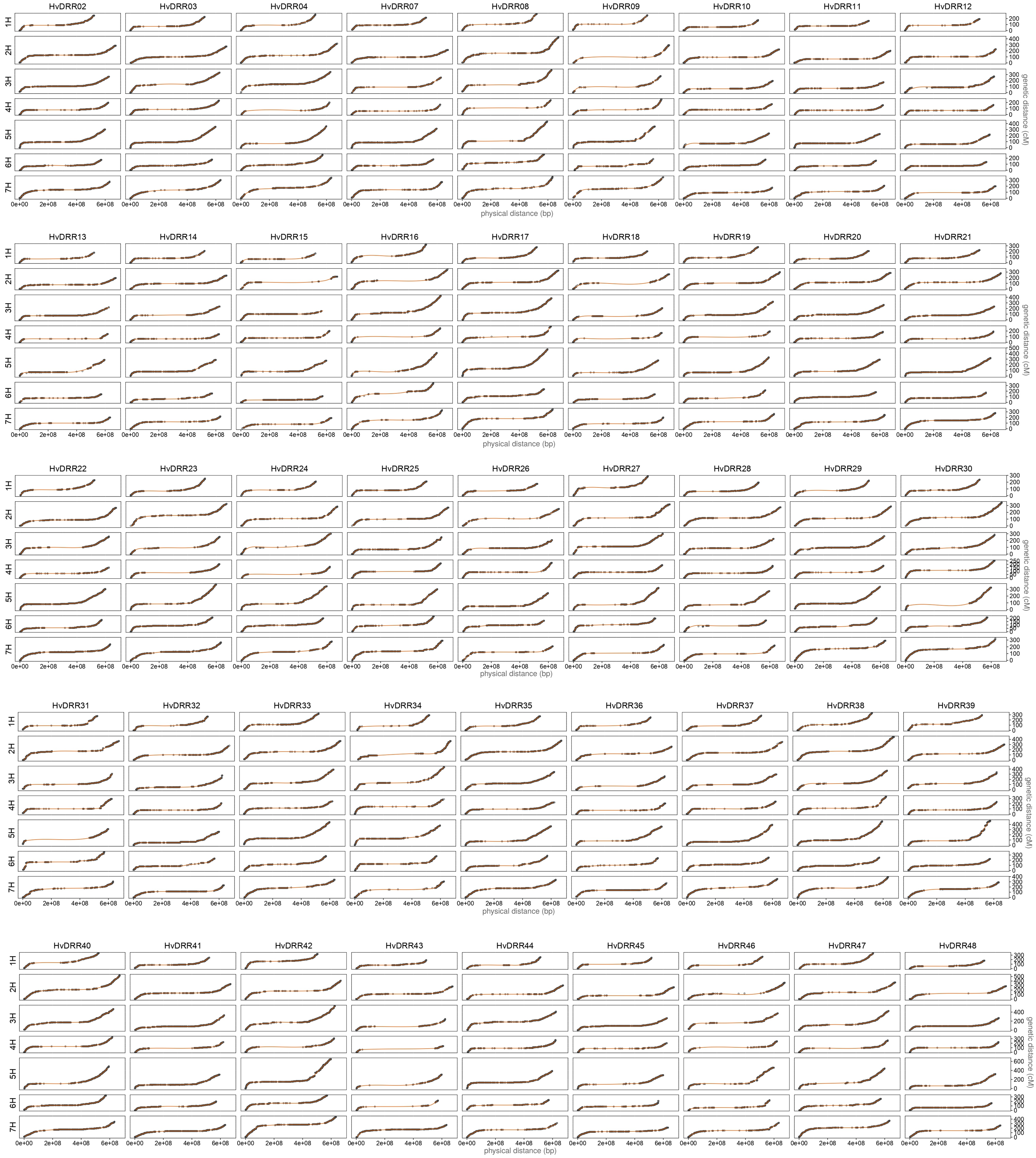

Fig. S3: The Marey maps of the seven barley chromosomes across the double round-robin populations.

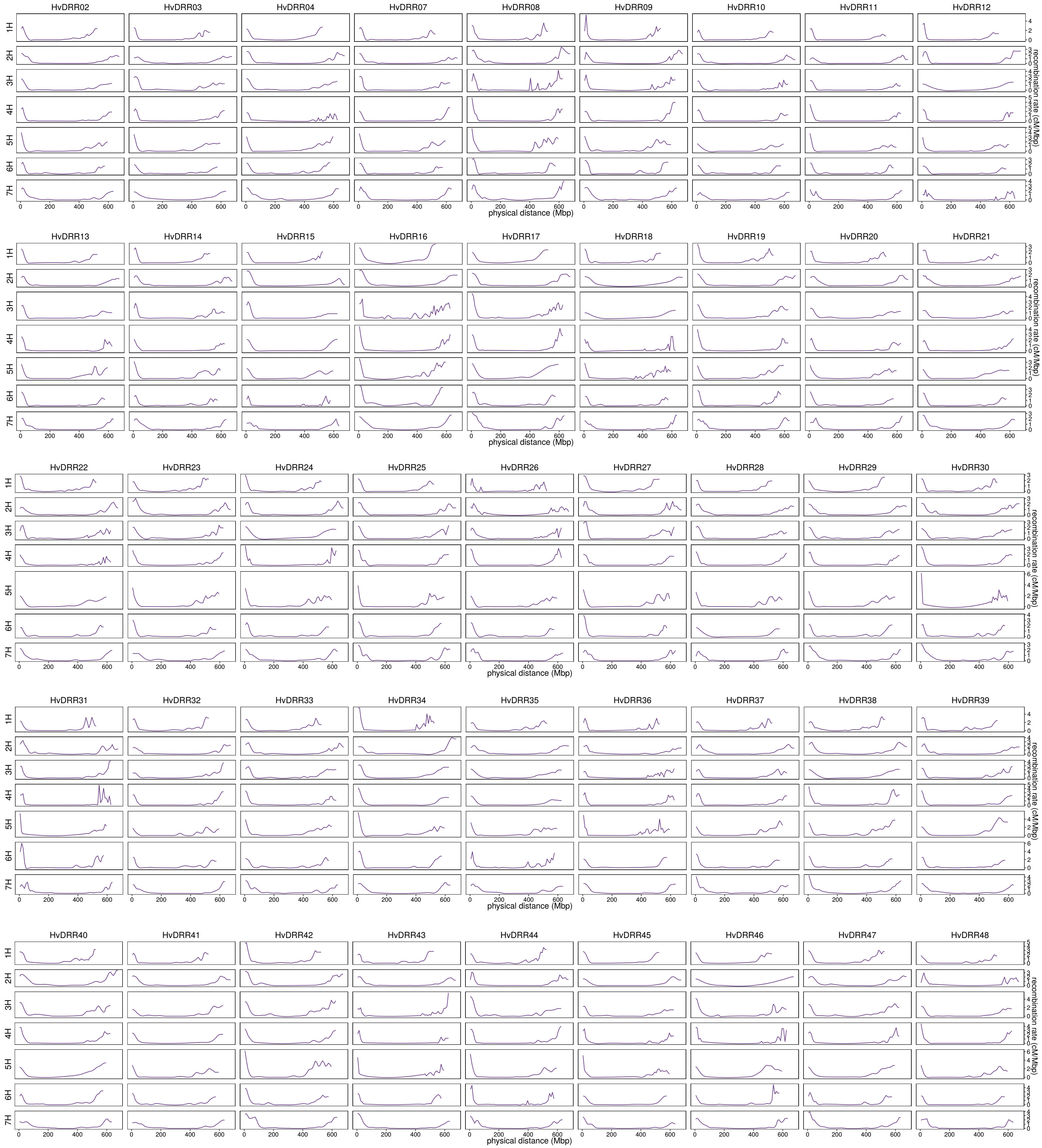

Fig. S4: The recombination landscape of the seven barley chromosomes across the double round-robin populations.

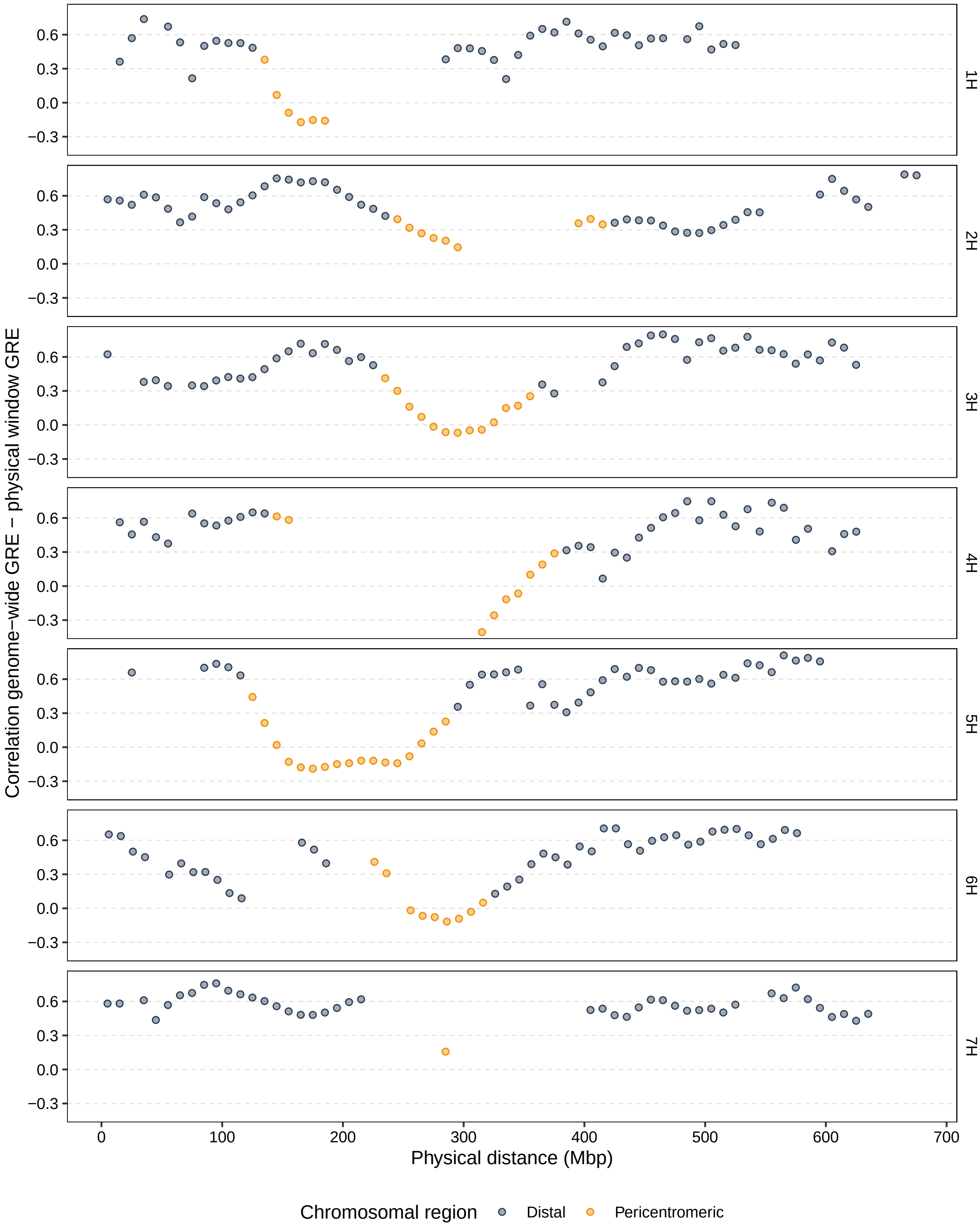

Fig. S5: Pearson's correlation coefficient between the GRE_P values of the parental inbreds for 10 Mbp physical windows and their respective genome-wide GRE_P values across the seven barley chromosomes.

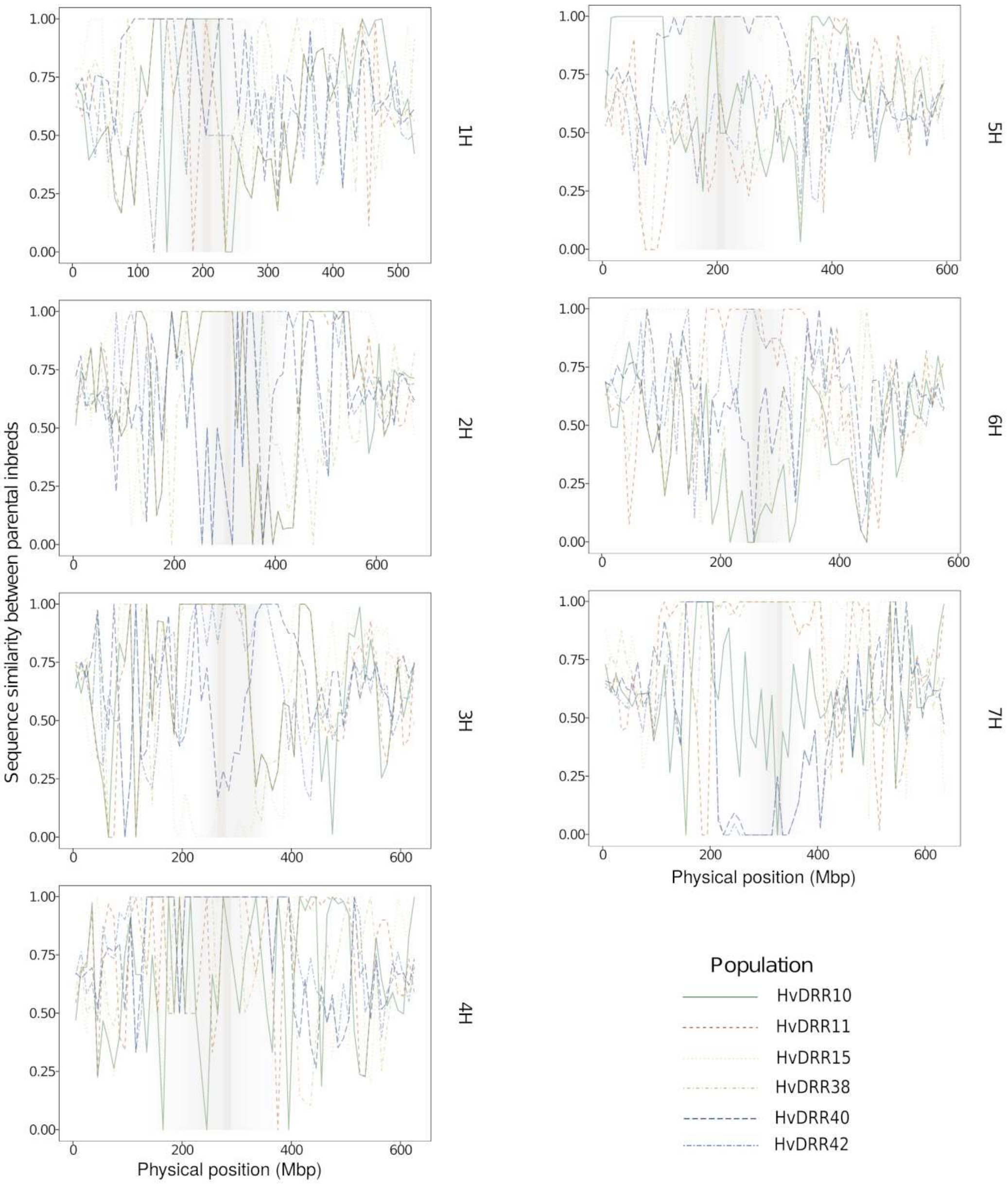

Fig. S6: The sequence similarity between the parental inbreds across the seven barley chromosomes of the three double round-robin populations with the highest and lowest genomic recombination rate. The vertical line in the background indicates the centromere position in the reference map, and the expanded shadow indicates the pericentromeric region in the consensus map.

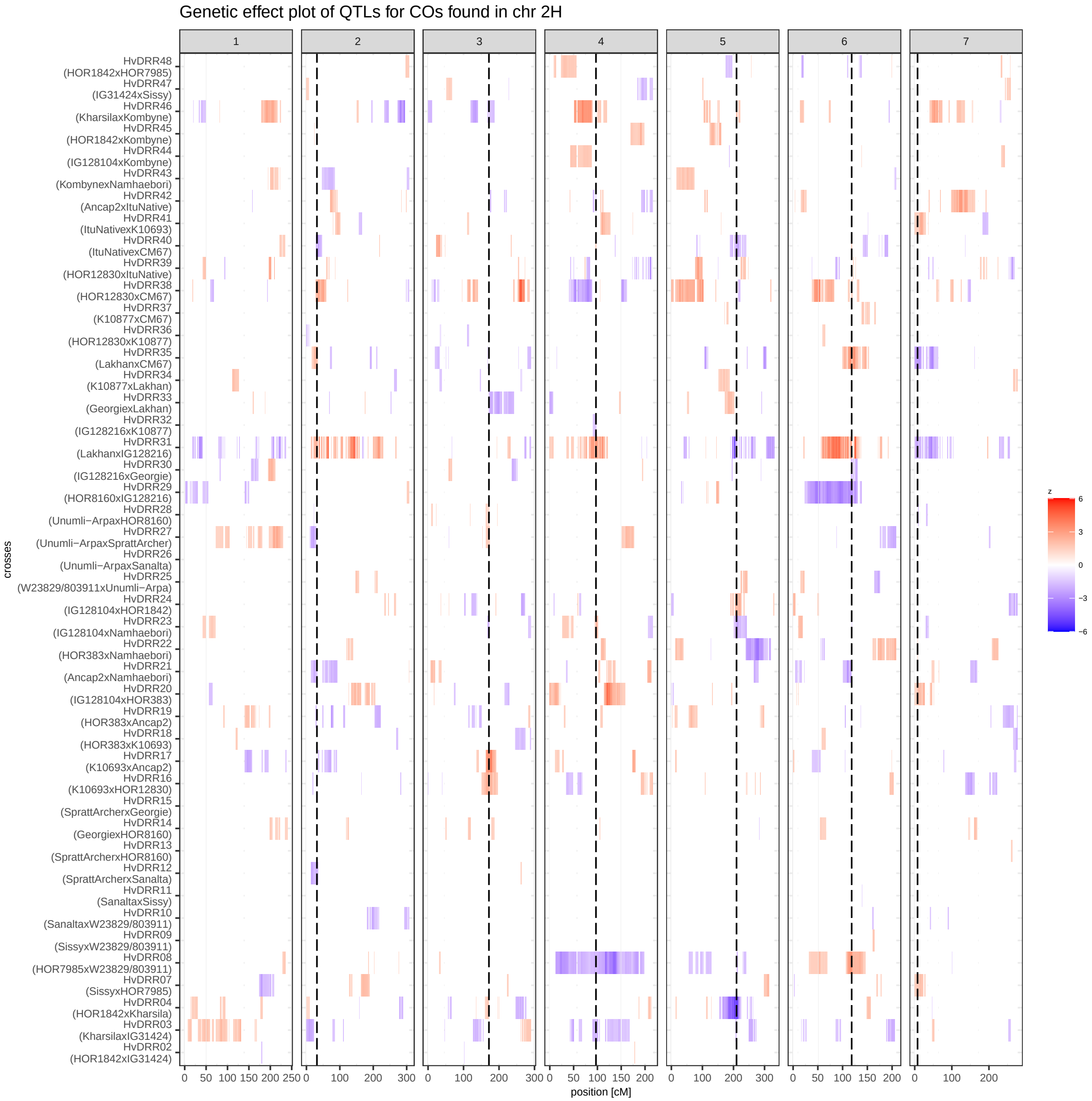

Fig. S7: The effect of the QTLs associated with the number of crossovers on chromosome 2H across the 45 double round-robin populations. The QTLs' locations are identified with a dashed black line.

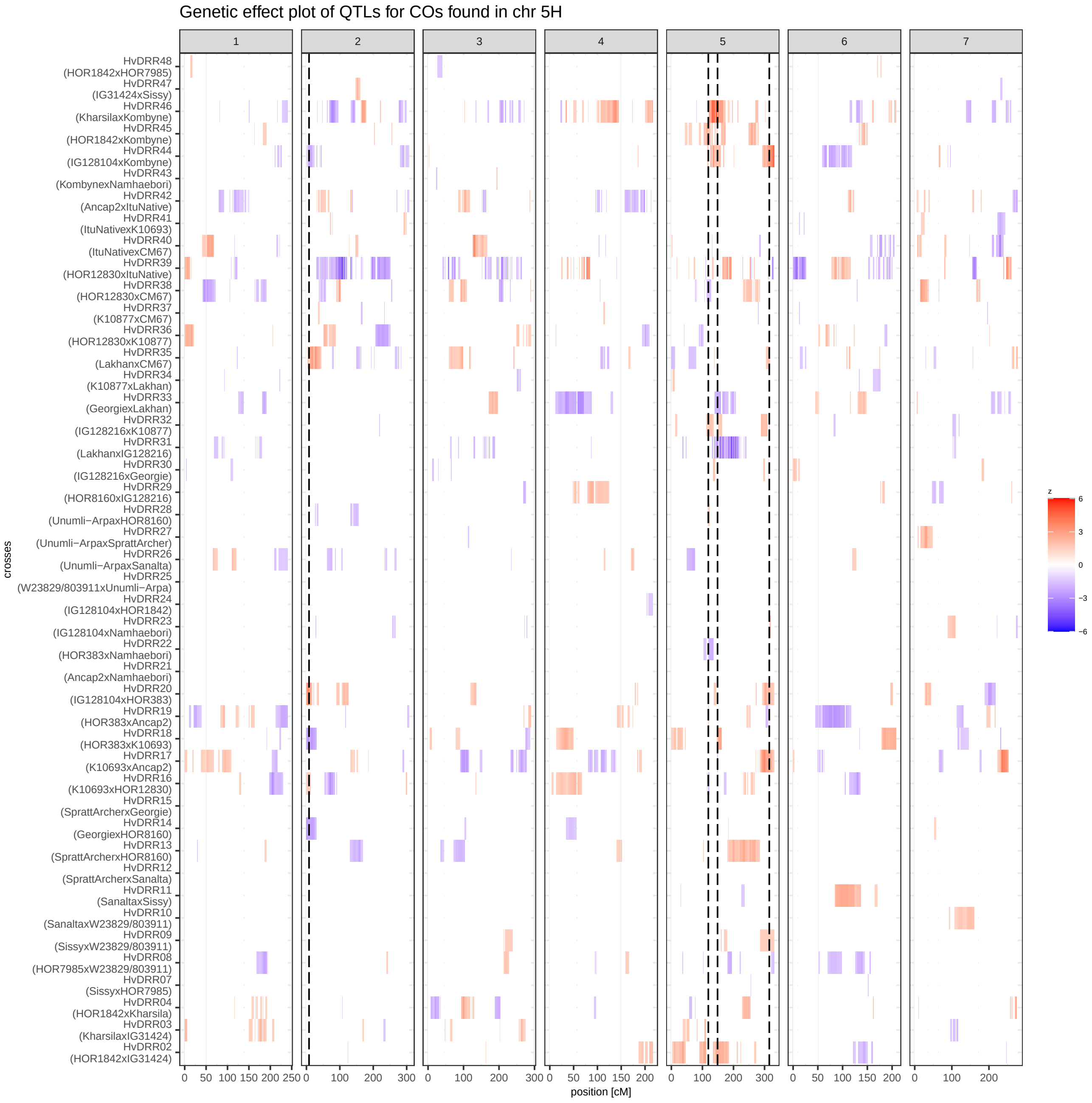

Fig. S8: The effect of the QTLs associated with the number of crossovers on chromosome 5H across the 45 double round-robin populations. The QTLs' locations are identified with a dashed black line.

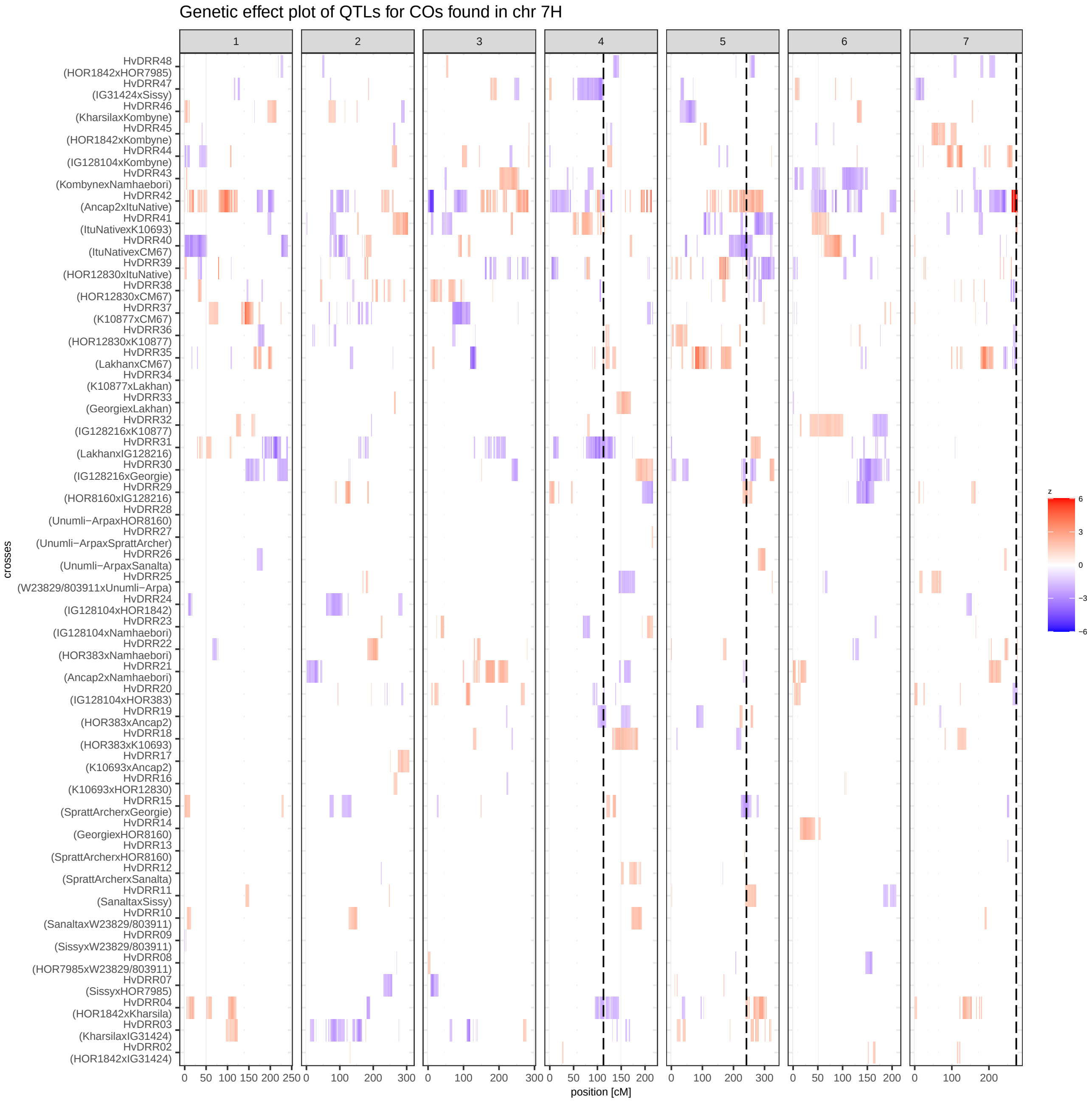

Fig. S9: The effect of the QTLs associated with the number of crossovers on chromosome 7H across the 45 double round-robin populations. The QTLs' locations are identified with a dashed black line.

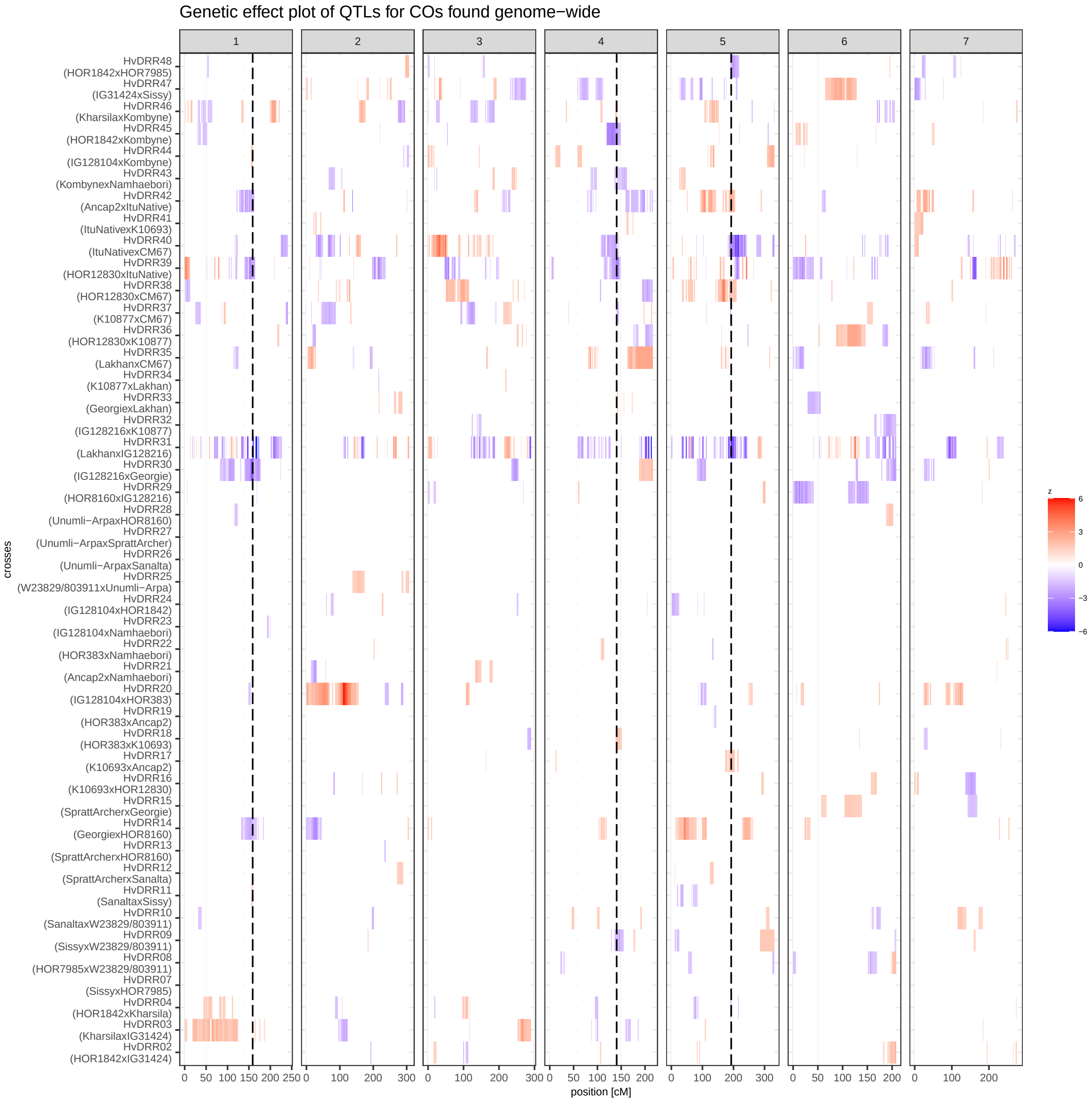

Fig. S10: The effect of the QTLs associated with the number of crossovers on the genome across the 45 double round-robin populations. The QTLs' locations are identified with a dashed black line.

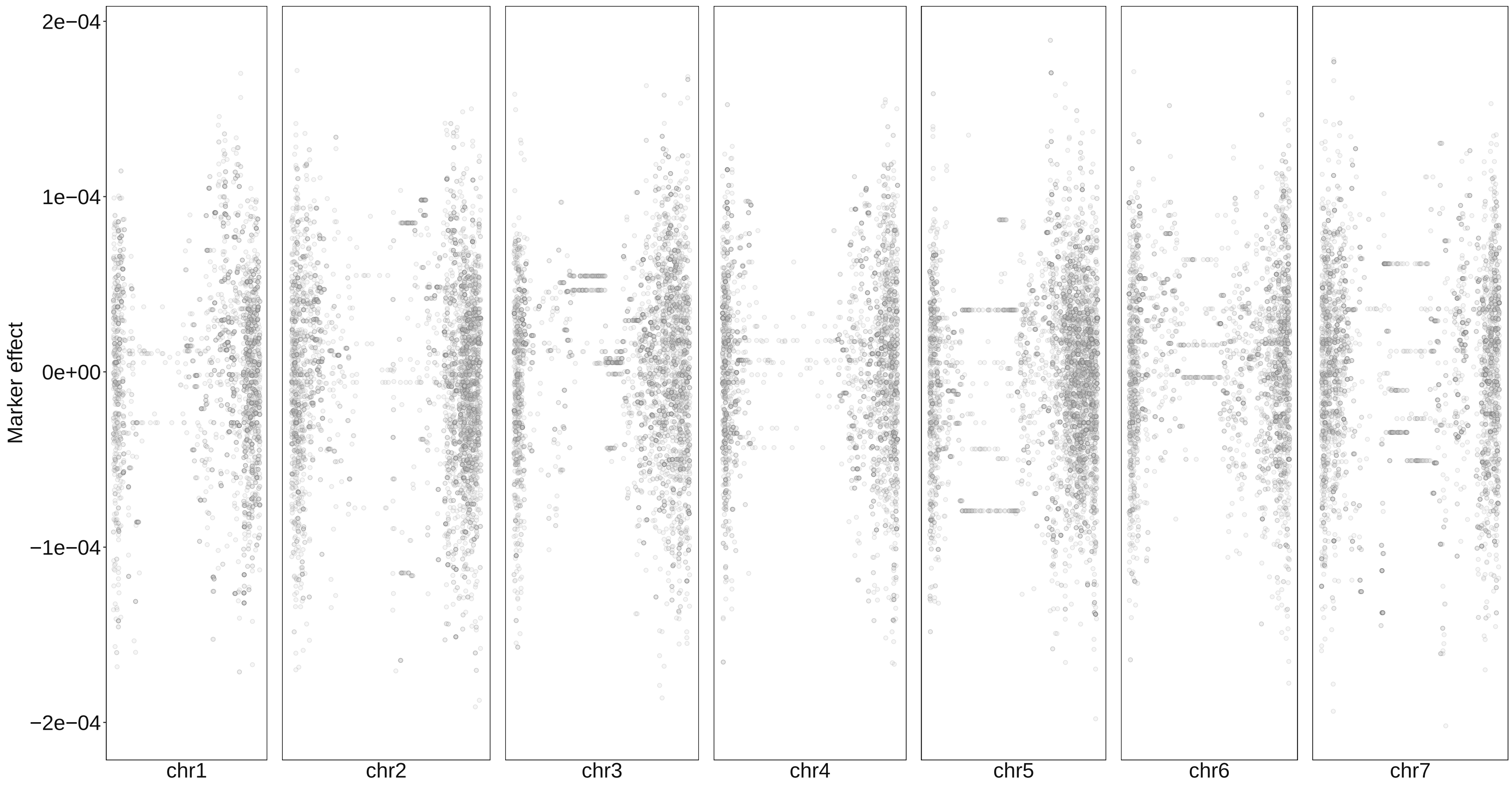

Fig. S11: The distribution of SNPs' effects predicted by RR-BLUP across the genome.

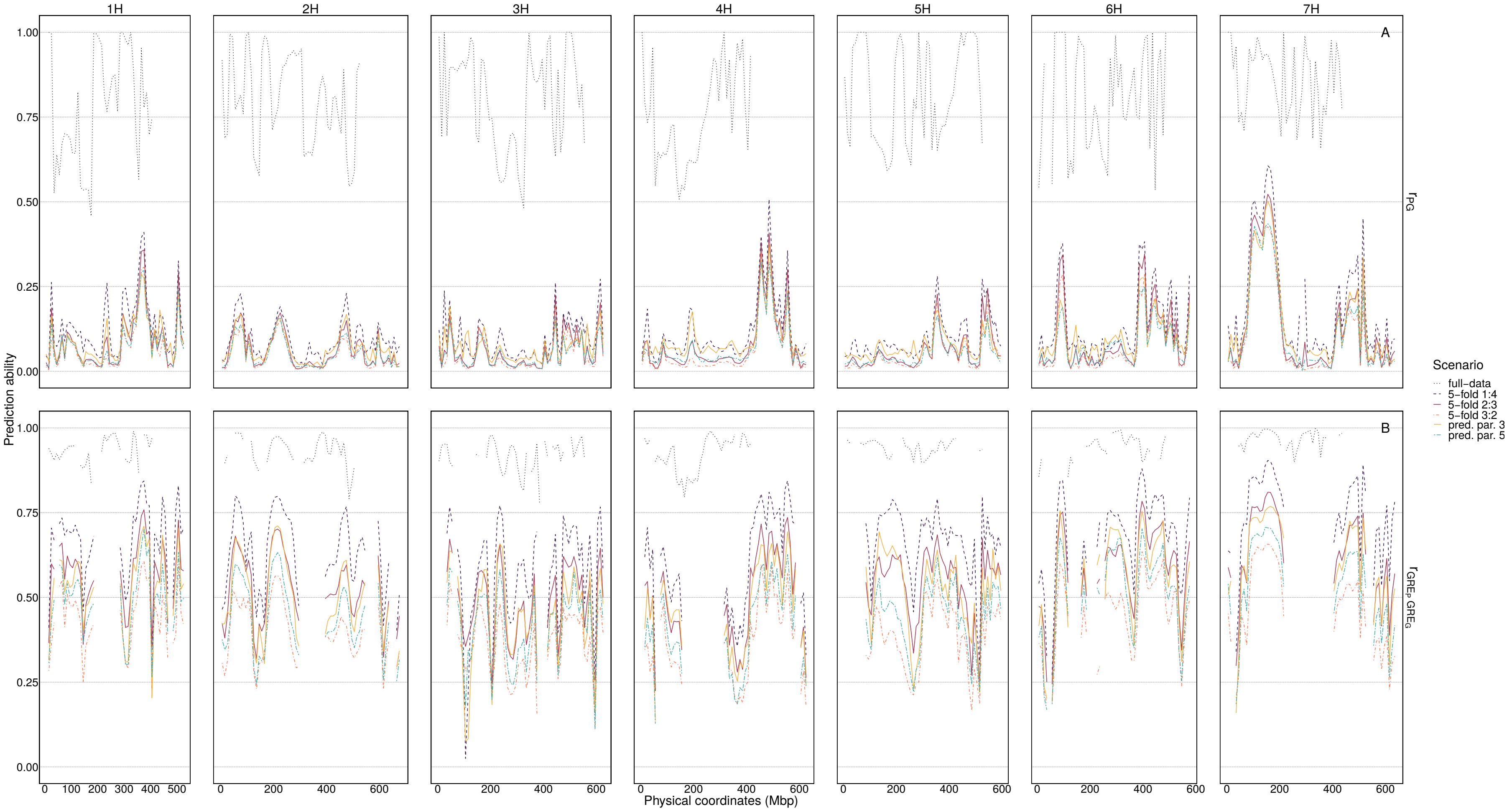

Fig. S12: The genomic prediction ability of recombination rate in 10 Mbp window level across the genome, using different cross-validation scenarios. (A) Pearson's correlation coefficient between the observed recombination rate and genomic estimated breeding values of the DRR populations, r_{PG}. (B) Pearson's correlation coefficient between the phenotypic and genomic estimated general recombination effects of the parental inbreds, $r_{GRE_P GRE_G}$. The cross-validation scenarios for genomic prediction are detailed in Figure 5.

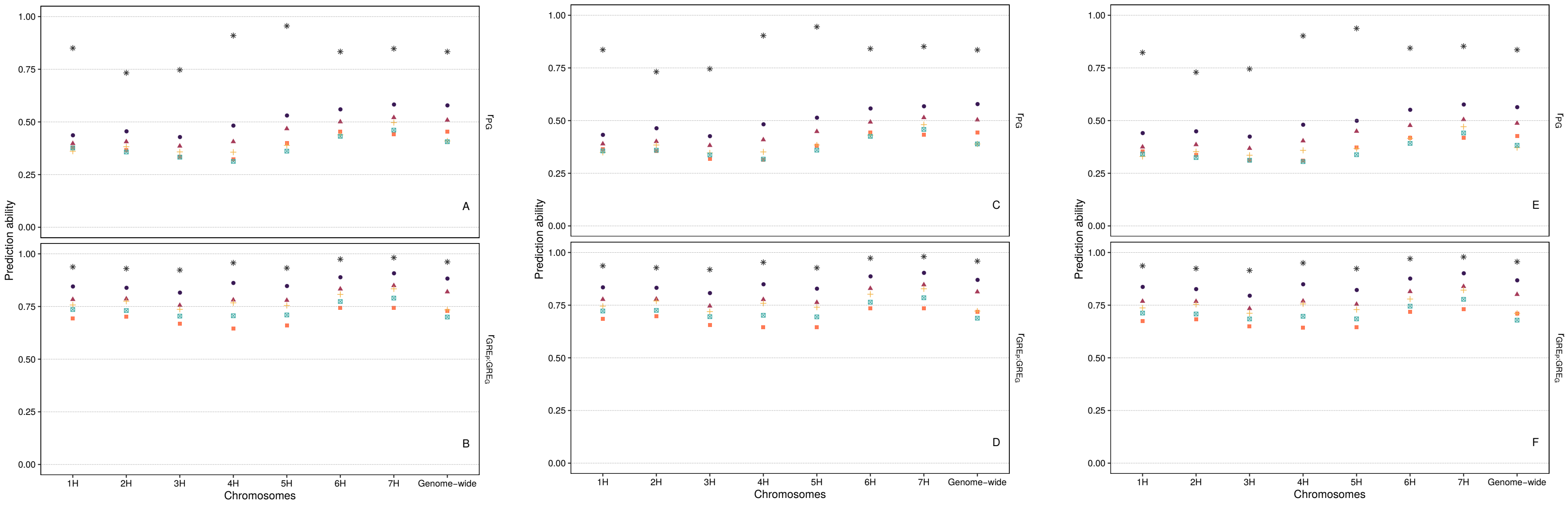

Fig. S13: Genomic prediction ability concerning the recombination rate variation of individual chromosomes and the genome-wide level, using different approaches and subsets of equally spaced SNPs. Genomic predictions in A and B were calculated using 1 SNP per 1 Mbp, in C and D using 1 SNP per 5 Mbp, and in E and F, using 1 SNP per 10 Mbp, respectively. (A, C, and E) Pearson's correlation coefficient between the observed recombination rate and genomic estimated breeding values of the DRR populations, r_{PG}. (B, D, and F) Pearson's correlation coefficient between the phenotypic and genomic estimated general recombination effects of the parental inbreds, $r_{(GRE_P GRE_G)}$. The cross-validation scenarios for genomic prediction are detailed in Figure 5.
